# Clinical validation of lightweight CNN architectures for reliable multi-class classification of lung cancer using histopathological imaging techniques

**DOI:** 10.1038/s41598-026-36652-6

**Published:** 2026-01-28

**Authors:** Ali Raza, Fareeha Hanif, Heba Abdelgader Mohammed

**Affiliations:** 1 Department of Mathematics, University of the Punjab, Lahore, Pakistan; 2 Department of Mathematics, University of Education, Vehari Campus, Vehari, Pakistan; 3 Technical and Engineering Specialties Unit, Applied College, King Khalid University, Mohyel Asser, Kingdom of Saudi Arabia

**Keywords:** Lung cancer classification, Histopathological images, Lightweight CNN, Digital pathology, Multi-class classification, Computational pathology, Model optimization, Cancer, Computational biology and bioinformatics, Oncology

## Abstract

Lung cancer remains one of the leading causes of cancer-related mortality worldwide, and accurate early diagnosis plays a critical role in improving patient survival. In this study, a comparative analysis of multiple lightweight Convolutional Neural Network (CNN) variants is presented for multi-class lung cancer classification using histopathological images. Four CNN architectures were designed to systematically explore the trade-off between model complexity and classification performance. Each variant was trained and evaluated within a unified experimental framework incorporating data augmentation, class balancing via computed class weights, and a custom macro-F1-based early stopping callback to ensure stable and fair performance comparison. The models were trained on three histopathological classes, Lung Benign Tissue, Lung Adenocarcinoma, and Lung Squamous Cell Carcinoma. The training process involved automated generation of accuracy, loss, and validation F1 curves, along with confusion matrices for both validation and test datasets. To assess robustness, the best-performing model was evaluated across multiple random seeds and statistical significance was established using paired McNemar’s tests against competing variants. Among the proposed variants, one model (Lite-V2) achieved superior macro-F1 performance and demonstrated strong generalization capability on unseen test data, confirming the effectiveness of lightweight CNNs in achieving high accuracy with reduced computational cost. This work highlights the potential of custom lightweight CNN architectures for efficient and reliable lung cancer classification, offering a reproducible framework that can be extended to larger datasets or adapted for clinical diagnostic applications.

## Introduction

Lung cancer is one of the most prevalent and deadly cancers worldwide. Accurate diagnosis from histopathological images is critical, as different subtypes like adenocarcinoma, squamous cell carcinoma, and small cell carcinoma require distinct treatment plans. Traditionally, this analysis is performed manually by pathologists, a process that can be time-consuming and subjective. Recent advances in deep learning, particularly Convolutional Neural Networks (CNNs), have shown great potential for automating the classification of medical images. Standard deep learning models like VGG and ResNet, while accurate, are computationally very expensive. Their large size and high processing demands make them difficult to deploy in real-world clinical settings with limited resources. To address this challenge, lightweight CNN variants such as MobileNet, EfficientNet, and ShuffleNet have been developed. These models are specifically designed to be efficient, using fewer parameters and computations while maintaining high accuracy. his paper presents a comprehensive evaluation of these lightweight CNN architectures for the multi-class classification of lung cancer from histopathological images. Our goal is to identify models that offer the best balance between classification performance and computational efficiency, making automated diagnosis more feasible for practical clinical use.

### Background

Lung cancer remains the leading cause of cancer-related mortality worldwide and poses substantial clinical challenges due to its heterogeneous presentation and often late-stage diagnosis. Early and accurate classification of lung lesions directly informs treatment planning, therapeutic selection, and prognosis; however, histopathological diagnosis requires extensive expertise and is time consuming, which can delay critical decisions^1,2^. Histopathological examination of hematoxylin and eosin (H&E)-stained tissue sections remains the clinical gold standard for definitive tumor typing and subtyping (for example, differentiating adenocarcinoma from squamous cell carcinoma)^3^, but inter-observer variability and the increasing case-load in many pathology departments motivate automated, reproducible decision-support tools.

Digital histopathology, where whole slide images (WSIs) or high-resolution image patches are digitized, enables computational analysis at scale and creates an opportunity for machine learning to augment human interpretation^4^. Traditional handcrafted feature methods have gradually been replaced by data-driven feature learners; in particular, Convolutional Neural Networks (CNNs) have achieved remarkable performance in visual pattern recognition tasks, including cancer detection and subtype classification from histology images. Recent works have demonstrated that CNNs and ensemble deep-learning pipelines can accurately discriminate lung cancer subtypes and extract prognostic information from H&E images without manual region-level annotations, enabling scalable analysis across large multi-institutional cohorts^5^. Despite strong performance, many high-performing networks are large, computationally expensive, and sometimes brittle when applied to images with different staining conditions or scanning devices. This reality motivates research into lightweight, efficient CNN designs that retain high classification performance while reducing memory footprint, inference time, and training cost, properties that are essential when integrating automated pathology tools into routine clinical workflows or deploying them on constrained hardware^6^. In parallel, recent large-scale studies and curated histopathology datasets have improved model development and external validation practices, enabling more reproducible and clinically relevant assessments of deep learning methods in lung pathology.

### CNNs in cancer detection

Convolutional Neural Networks (CNNs) have become foundational models in computer vision and have been extensively adopted in medical imaging, resulting in substantial advances in automated cancer detection. Unlike classical image processing pipelines that rely on handcrafted features (such as texture descriptors, shape features, or color histograms), CNNs learn hierarchical and task-specific representations directly from raw pixels, progressively transforming local patterns into global abstractions. This end-to-end learning capability allows CNNs to capture subtle morphological variations in tissue, cellular architecture, and staining patterns that are difficult to encode manually, thereby improving sensitivity and specificity in diagnostic tasks. In cancer pathology, CNNs have been used to address a spectrum of tasks including tumor region segmentation, subtype classification, biomarker prediction, and survival prognosis. For instance, recent reviews on deep learning applications in clinical cancer detection highlight how CNN-based models achieved high accuracy across radiology and pathology modalities, enabling early detection, subtype differentiation, and molecular inference from histological images^7^. In lung cancer specifically, CNNs have been applied to classify adenocarcinoma versus squamous cell carcinoma and even small cell lung carcinoma using H&E-stained slides or cytological specimens^8^. These models have shown promise in assisting pathologists, reducing diagnostic burden, and improving throughput.

**Fig. 1:**
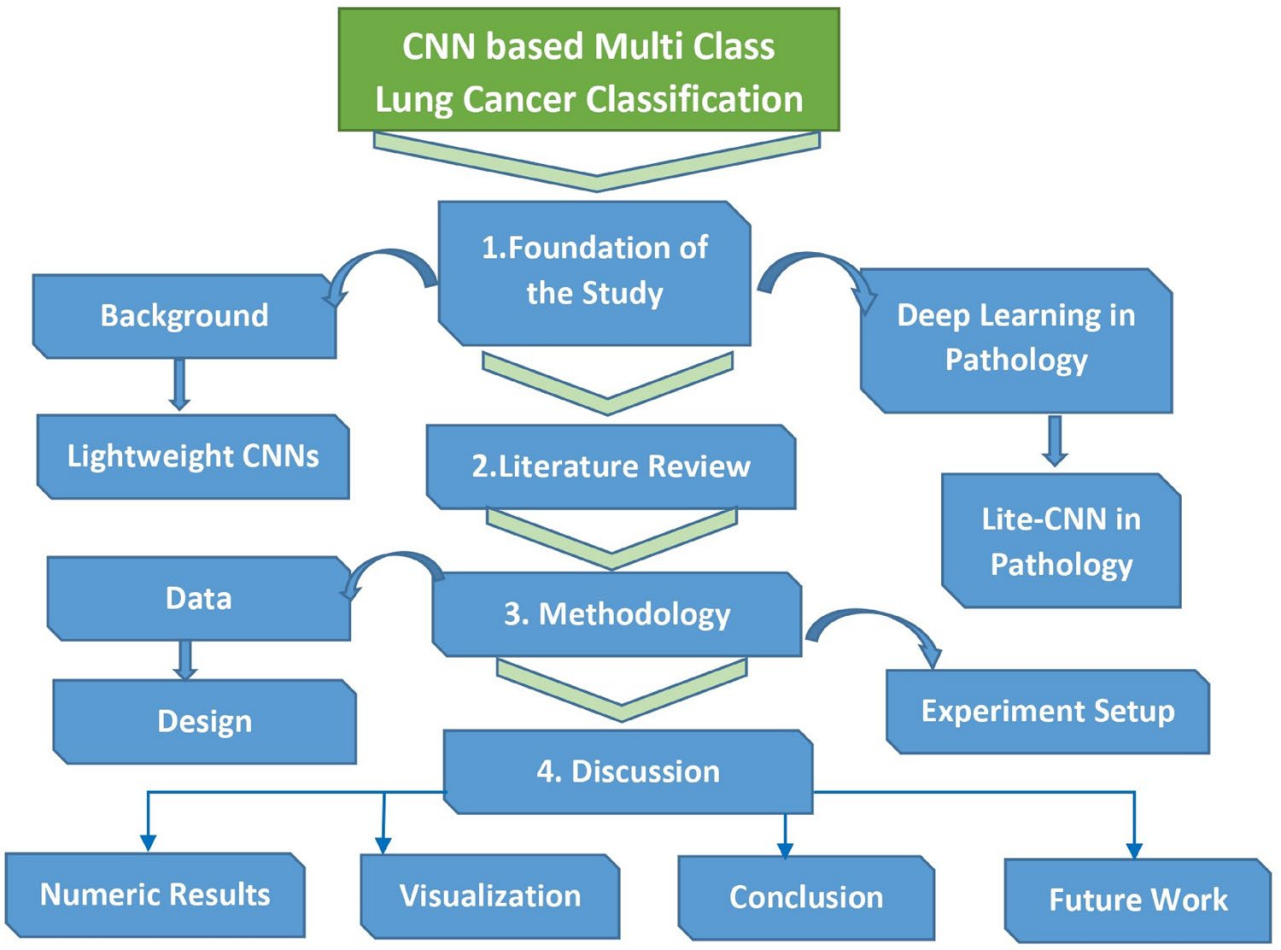
Hierarchical representation of the proposed article structure illustrating the interconnections among key components and methodological layers.

Many studies adopt classical CNN backbones initially developed for natural image classification, such as VGG, ResNet, Inception, DenseNet, and their variants, often leveraging transfer learning to adapt pretrained weights to medical image domains. For example, Baranwal et al. used variants such as ResNet-50, VGG-19, and Inception-ResNet-V2 to classify lung histopathology into benign, adenocarcinoma, and squamous carcinoma, showing competitive performance across architectures^9^. Comparative analyses of deep models on histopathologic cancer detection datasets (e.g. comparing ResNet50, VGG16, InceptionV3, DenseNet, MobileNet, Xception) reveal notable differences in classification performance, computational cost, and robustness under stain variation or domain shift^10^. Such studies often highlight trade-offs between model complexity and clinical utility. The complete structure of our article is presented in the Figure 1.

Recent advances in medical image analysis have increasingly emphasized not only predictive accuracy but also model transparency, interpretability, and multimodal understanding, particularly due to the high-stakes nature of clinical decision-making. Mir and Rizvi^11^ provide a comprehensive and up-to-date survey on deep learning and explainable artificial intelligence (XAI) in medical imaging, systematically categorizing explainability techniques into visual, textual, example-based, and concept-based paradigms. Their work highlights that despite strong performance, many deep learning models remain difficult to integrate into real clinical workflows due to their black-box nature, underscoring the necessity of explainability-aware architectures and standardized evaluation metrics. Building upon this direction, Mir et al.^12^ propose an Explainable Vision Transformer (XViT) tailored for histopathological image analysis, combining high classification accuracy with robust interpretability through attention-based, gradient-based, and model-agnostic explanation mechanisms. Their extensive evaluation on large-scale histopathology datasets demonstrates that transformer-based models can achieve both strong diagnostic performance and clinically meaningful explanations when coupled with appropriate interpretability metrics. Beyond image-level classification, recent studies have also explored multimodal learning to bridge visual analysis and clinical reporting. In this context, Mir et al.^13^ introduce a self-boosting multimodal alignment framework that integrates vision transformers with language models for automated medical image report generation, achieving significant improvements on benchmark datasets such as IU-Xray and MIMIC-CXR. Collectively, these studies reflect a clear shift in the field toward explainable, trustworthy, and multimodal medical imaging systems. However, many of these approaches rely on computationally heavy architectures, motivating the need for efficient and lightweight models that balance performance, interpretability, and deployability in real-world clinical environments. However, large and deep CNNs face several limitations when directly deployed in medical imaging tasks:

1. Computational cost and memory footprint: Architectures such as ResNet-152 or Inception-ResNet require substantial GPU memory, long training times, and high inference latency, which hinder deployment in resource-constrained clinical settings or real-time workflows.
2. Overfitting and limited generalization: Medical imaging datasets are often relatively small and highly imbalanced. Deep networks tend to overfit to training distributions or stain protocols, degrading performance when applied to external institutions or different imaging equipment^14^.
3. Domain shift and robustness: Variations in slide preparation, staining, scanning, and inter-laboratory differences introduce domain shifts, which can lead to performance drop in models that lack domain adaptation or robust regularization strategies^15^.
4. Interpretability and regulatory concerns: CNNs often act as “black boxes,” making it difficult for clinicians to understand decision rationale. This hinders clinical acceptance, validation, and regulatory approval processes.

Because of these bottlenecks, recent research has increasingly focused on designing lightweight and efficient CNN architectures or hybrid models that balance accuracy and resource constraints. Some works also incorporate methods such as stain normalization, data augmentation, ensemble strategies, attention modules, and lightweight backbone designs to mitigate overfitting and improve robustness. Such approaches aim to make CNN-based cancer classification more practical for real-world, cross-center diagnostic applications.

### Motivation

The development of custom lightweight CNNs in this work is motivated by three practical and methodological needs that arise when translating deep learning research into routine histopathology practice: (1) the requirement for simpler, lighter, and faster models that are suitable for limited computational resources and real-time or near-real-time clinical pipelines; (2) the desire to evaluate multiple model variants under identical experimental conditions so that architecture-level trade-offs can be meaningfully compared; and (3) the importance of optimizing and monitoring balanced performance (macro-F1) rather than relying solely on accuracy when class distributions are imbalanced or when per-class performance matters clinically. Each motivation is described in detail below with supporting evidence from recent literature.

#### Need for simpler, lighter, and faster models

Deploying deep-learning models in pathology laboratories or at the edge (e.g., in low-resource hospitals or on embedded devices) imposes strict constraints on memory, inference latency, and compute throughput. Large backbones (e.g., ResNet-152, Inception-ResNet) can achieve high accuracy but are expensive to train and slow to run in inference mode, which complicates integration with laboratory information systems and rapid diagnostic workflows. Recent studies therefore emphasize the design of lightweight, parameter-efficient architectures that preserve strong classification performance while reducing computational footprint. Examples include multi-scale lightweight CNN proposals and extremely lightweight networks tailored for lung/colon histopathology that achieve competitive performance with substantially fewer parameters and faster inference times^16–18^. Lightweight architectures also facilitate systematic hyperparameter search and multiple-run experiments (necessary for robust evaluation) because each training run consumes fewer resources and completes faster.

#### Comparative evaluation under identical conditions

A core motivation behind this study is the need to compare multiple handcrafted CNN variants (Lite-V0, Lite-V1, Lite-V2, Lite-V4) under the same preprocessing, augmentation, training, and evaluation pipeline. Comparative evaluation under identical conditions reduces confounding factors and clarifies whether observed performance differences arise from architecture design or from dataset/optimization differences. Recent benchmarking efforts and comparative analyses in histopathology advocate for reproducible experimental pipelines, standardized splits, and logging of model checkpoints and run metadata, because ad-hoc comparisons across different studies and preprocessing pipelines can be misleading^19,20^. Lightweight models make such multi-run comparative studies practical by lowering the cumulative compute required to explore architectural variants and hyperparameters.

#### Importance of F1-based validation rather than only accuracy

Accuracy alone can be misleading in multi-class or class-imbalanced medical datasets because it weights each sample equally and can obscure poor performance on less frequent but clinically important classes. The macro-averaged F1 score (macro-F1) treats each class equally by computing the unweighted mean of per-class F1 scores, providing a balanced measure that is sensitive to both precision and recall for each label. Consequently, optimizing and monitoring macro-F1 during training leads to models that maintain balanced performance across classes, an important property when misclassifying a rarer but clinically significant tumor subtype has severe consequences. Several recent works and practical guides recommend using macro-F1 (or class-weighted metrics) for early stopping and model selection in medical image classification tasks; they also emphasize implementing reliable, reproducible monitoring (e.g., callbacks that compute macro-F1 on validation folds) rather than relying solely on validation loss or accuracy as stopping criteria^21–23^. In this study we therefore adopt a macro-F1-based callback that saves the best model according to validation macro-F1 and triggers early stopping when macro-F1 plateaus, which encourages balanced generalization across all lung tissue classes.

#### Clinical relevance and deployment readiness of lightweight CNNs

Beyond technical performance, clinical deployment of AI models for lung cancer histopathology requires explainability, efficiency, and integration readiness. Lightweight CNNs that maintain transparency (through simpler layer structures) and require fewer computational resources are easier to audit and validate within regulated medical environments. Additionally, smaller models can be deployed locally on hospital servers or embedded systems without cloud dependency, ensuring patient data privacy and compliance with medical data governance frameworks such as HIPAA and GDPR^24^. Therefore, lightweight architectures not only address engineering efficiency but also advance the clinical feasibility of AI-assisted diagnostic tools.

### Objective and contributions

This study sets out to design, implement, and evaluate lightweight Convolutional Neural Network (CNN) variants for multi-class lung cancer classification using histopathological images. The objectives combine practical deployment goals (efficiency, low-latency inference) with methodological rigor (reproducible comparisons and balanced metrics). The primary contributions of the work are listed below in an enumerated form; for each numbered objective we provide a short bulleted breakdown that clarifies the sub-tasks and intended deliverables as Table1.

1. Develop and compare multiple lightweight CNNs.
  (i) Design four compact CNN variants (Lite-V0, Lite-V1, Lite-V2, Lite-V4) that differ in depth and filter counts to explore accuracy–efficiency trade-offs.
  (ii) Implement each architecture as a modular function so models can be instantiated, trained, and evaluated with identical data pipelines.
  (iii) Benchmark parameter counts, FLOPs (if measured), training time, and inference latency in addition to classification performance to quantify computational benefits versus accuracy loss.
  (iv) Rationale and precedent: recent studies have shown that carefully engineered lightweight networks can achieve competitive histopathology classification while dramatically reducing compute requirements, making them suitable for deployment in resource-constrained clinical environments^27^.
2. Design an F1-based early stopping strategy.
  (i) Implement a custom training callback that computes macro-averaged F1 on the validation set at the end of each epoch.
  (ii) Save model checkpoints according to the best validation macro-F1 and trigger early stopping when macro-F1 stagnates for a configurable patience period.
  (iii) Justification: macro-F1 is a balanced metric for multi-class and imbalanced datasets; prior works and practical recommendations endorse F1-based monitoring for clinical classification tasks^25,28^.
3. Generate accuracy/loss/F1 plots and confusion matrices.
  (i) Automatically log and plot training and validation accuracy and loss across epochs for each run.
  (ii) Plot validation macro-F1 versus epoch to visualize balanced class performance during training.
  (iii) Compute confusion matrices and classification reports (precision, recall, F1 per class) for validation and test splits to diagnose class-specific errors and guide model selection.
  (iv) Visual diagnostics enhance interpretability and are recommended by reproducibility checklists in medical imaging AI^26^.
4. Identify the best performing variant.
  (i) Rank models primarily by validation macro-F1 and secondarily by validation accuracy and computational cost.
  (ii) Evaluate the top-ranked model on an independent test set and report per-class metrics, confusion matrix, and qualitative error analysis.
  (iii) Use saved checkpoints and run logs to ensure that the chosen model is reproducible and its performance can be independently verified^29^.
5. Present the results in a reproducible, modular experiment setup.
  (i) Provide a modular codebase structure: data loaders, augmentation pipelines, model builders, training runner, callbacks, plotting utilities, and results logger (JSON).
  (ii) Save experiment metadata (random seed, dataset split identifiers, hyperparameters, best checkpoint path) for each run to facilitate replication and ablation studies.
  (iii) Follow reproducibility and reporting recommendations recently proposed for deep learning in medical imaging to maximize transparency and reusability.

**Table 1:** Summary of core research objectives, key contributions, and corresponding methodological solutions.

Objective	Key contributions/Sub-tasks	Representative citations
Develop lightweight CNNs	Design Lite-V0 to Lite-V4 variants; implement modular architectures; benchmark efficiency vs. accuracy; justify lightweight approach	[^86^]
F1-based early stopping	Macro-F1 computation; checkpoint saving; early stopping; justification for multi-class balance	[^49^]
Plot metrics & confusion matrices	Accuracy/loss/F1 plots; class-specific confusion matrices; visual diagnostics for interpretability	^25^
Identify best variant	Model ranking by macro-F1; evaluate on test set; per-class metrics and qualitative analysis	^26^
Reproducible modular setup	Modular codebase; save experiment metadata; ensure replication and transparency	[^18^]

## Related work

### CNNs in lung cancer detection

Deep learning, particularly Convolutional Neural Networks (CNNs), has revolutionized computer-aided diagnosis in pulmonary oncology by enabling automatic detection, segmentation, and classification of cancerous lesions across multiple imaging modalities. This section summarizes key advancements in the application of CNNs to lung cancer detection using computed tomography (CT), X-ray imaging, and histopathological images, as well as hybrid and transfer learning approaches that enhance performance and generalization.

#### CNNs in CT-based lung cancer detection

CNNs have become foundational in the analysis of CT scans for early lung cancer screening and nodule classification. Several 2D and 3D CNN frameworks have been employed for identifying and characterizing pulmonary nodules in low-dose CT (LDCT) scans. Recent works demonstrate that deep 3D CNN architectures can achieve sensitivity above 90% for nodule detection, outperforming classical computer vision methods^30^. Researchers have also integrated CNNs into the diagnostic pipeline for malignancy prediction, incorporating volumetric analysis and morphological features of nodules. A 2023 study employed a multi-scale 3D ResNet architecture on the LIDC-IDRI dataset, reporting an AUC of 0.94 for benign–malignant classification^31^. Similarly, transfer learning from ImageNet-pretrained CNNs such as VGG and ResNet improved performance when adapted to CT slice classification, especially under limited labeled data conditions^32^. However, challenges persist in domain shift, model interpretability, and robustness across scanners and institutions, which limit widespread clinical deployment.

#### CNNs in X-ray image analysis

In X-ray imaging, CNNs have shown promising results for screening and early detection of lung malignancies. Large public datasets such as X-ray14 and CheXpert have facilitated the training of CNNs capable of recognizing lung abnormalities indicative of cancerous lesions. A 2024 study introduced a transfer-learning-based ResNet-50 model fine-tuned on lung cancer subsets of X-ray14, achieving 96.2% accuracy in distinguishing malignant from normal scans^33^. Lightweight CNN architectures have also been explored to reduce computational costs and enable on-device inference, which is crucial for screening in resource-limited healthcare settings. For example, an EfficientNet-B0 model trained on augmented X-ray images reported comparable performance to heavier backbones with a fraction of parameters^34^. Despite strong detection results, X-ray-based CNNs often face limitations due to overlapping tissue structures, variable image quality, and the lack of 3D contextual information.

#### CNNs in histopathology-based lung cancer classification

CNNs have also achieved major breakthroughs in analyzing histopathological images, where cancer diagnosis depends on recognizing cellular morphology and tissue architecture. Early efforts utilized handcrafted CNNs such as VGG-19 and ResNet-50 for classifying hematoxylin and eosin (H&E)-stained image patches into benign, adenocarcinoma, and squamous cell carcinoma categories. More recently, advanced CNN models such as EfficientNet and DenseNet variants have shown superior performance in lung histopathology classification, with reported accuracies above 98% on benchmark datasets such as LC25000 and LungHist700^35^. In addition, explainable deep learning frameworks have been developed to highlight discriminative tissue regions, improving interpretability and clinical trustworthiness^36^. Recent hybrid CNN-transformer architectures further enhance feature extraction by capturing long-range tissue dependencies within histopathology slides^37^. Nevertheless, computational complexity and data imbalance remain significant challenges in large-scale histopathological classification.

#### Transfer learning and hybrid deep learning approaches

Transfer learning and hybrid deep learning strategies have become increasingly important in lung cancer detection. By fine-tuning pretrained models such as ResNet, Inception-V3, and EfficientNet, researchers have achieved robust performance even on relatively small medical datasets.

(i) CNN models pretrained on ImageNet and later fine-tuned on histopathology datasets outperformed scratch-trained models by more than 7–10% in overall accuracy^38^.
(ii) Hybrid approaches integrate CNN feature extractors with Support Vector Machines (SVMs) or transformer-based encoders to boost discriminative power and interpretability.
(iii) A 2025 study demonstrated that a CNN–Vision Transformer (ViT) hybrid achieved state-of-the-art results on lung biopsy slides with improved generalization across stain variations^39^.

These advancements underscore the potential of combining efficient CNN feature extraction with transformer-level contextual awareness for accurate and scalable lung cancer classification. Overall, CNN-based architectures, spanning CT imaging, X-ray, and histopathology, have achieved remarkable progress in automated lung cancer detection. Yet, they remain constrained by computational complexity, data heterogeneity, and limited cross-institutional generalization. The present study addresses these gaps by developing and comparing multiple lightweight CNN variants trained under identical experimental conditions, emphasizing balanced (macro-F1) evaluation and reproducibility across model configurations.

### Lightweight and custom CNNs

As deep neural networks become deeper and wider, their computational demands rise accordingly, presenting obstacles for deployment in real-world or resource-limited settings. In response, the research community has increasingly turned to lightweight CNNs and custom architectures optimized for efficiency. This section reviews five thematic strands of that trend: (i) MobileNet and depthwise separable convolution, (ii) EfficientNet scaling and compound model design, (iii) domain-specific and custom lightweight networks, (iv) deployment on edge or mobile hardware, and (v) pruning, quantization, and hybrid compression strategies for CNNs in medical imaging.

#### MobileNet and depthwise separable convolution

MobileNet is one of the foundational efficient CNN families, built around depthwise separable convolution to drastically reduce parameters and computation while retaining representational power. Its variants (MobileNetV1/V2/V3/V4) have been widely adopted in on-device and embedded vision tasks^40^. In medical imaging, the MobileNet backbone has been used in lightweight transfer-learning pipelines for X-ray, ultrasound, and histopathology classification, showing that a lightweight backbone can approach performance of larger networks with far fewer parameters^41^. For example, Ogundokun et al. proposed a MobileNet-SVM hybrid for breast imaging tasks that achieves good accuracy with low computational cost^42^.

#### EfficientNet and compound scaling

EfficientNet introduced the idea of compound scaling, balancing depth, width, and image resolution using a fixed scaling coefficient, to generate a series of models (EfficientNet B0 to B7) that maximize efficiency. In medical imaging, EfficientNet variants have been successfully employed for histopathology and radiology tasks, offering excellent trade-offs between accuracy and compute. A review of CNN methods in medical imaging notes that EfficientNet variants often outperform heavier backbones when dataset size is limited^43^. In histopathology specifically, EfficientNet has been used as a backbone for lung/colon image classification with favorable performance.

#### Domain-specific and custom lightweight CNNs

Beyond adapting mobile architectures, many works propose custom lightweight CNNs tailored to the particular domain (e.g. pathology textures, stain variation). For instance, ELW-CNN is an extremely lightweight CNN designed for cancer classification in histopathology images, achieving strong classification performance with minimal parameter overhead. Another example is ReducedFireNet, a compact design for histology image classification intended for IoMT (Internet of Medical Things) deployment, which reported a mean accuracy of 96.88 % and F1 = 0.968 with very small model size^44^. MobiHisNet is yet another custom lightweight CNN built for histopathological image classification and deployed on devices like Raspberry Pi and mobile phones^45^. Such domain-custom designs often outperform generic lightweight models by focusing on domain-specific feature patterns.

#### Edge and mobile deployment of lightweight models

(i) One of the principal motivations for lightweight CNNs is enabling **on-device inference** in constrained environments such as clinics, portable scanners, or even smartphones.
(ii) Studies have demonstrated deployment of CNNs on embedded hardware (e.g. NVIDIA Jetson, Raspberry Pi) for real-time medical image tasks^46^.
(iii) In pathology, MobiHisNet has been deployed to mobile devices to classify histology slides locally, reducing latency and preserving data privacy.
(iv) Recent proposals such as LACE-Net also emphasize lightweight architectures explicitly for histopathology image analysis with deployment in mind^47^.

#### Pruning, quantization, and hybrid compression in medical CNNs

1. Beyond architecture design, another trend is **post-training compression techniques** such as pruning (removing redundant weights) and quantization (reducing bit precision), or hybrid methods combining architecture design + compression.
2. These methods reduce model size and latency with minimal accuracy loss.
3. In medical imaging, pruning and quantization have been used to adapt heavier CNNs to constraints while maintaining diagnostic performance^48^.
4. Hybrid methods combining a lightweight CNN architecture with quantization or sparse representations show promise in reducing memory footprint further, making models more deployable in clinical hardware.
5. Overall, the trend toward lightweight and custom CNNs is well supported by both general-purpose and domain-specific medical imaging literature.
6. These techniques inform and justify our design of the Lite-V0 to Lite-V4 variants, aiming for efficient yet accurate classification in lung cancer histopathology.

### Identified gaps

Although deep learning has revolutionized computational pathology and lung cancer detection, several limitations remain evident in existing research. A major issue concerns the continued dominance of large-scale pretrained networks such as ResNet, DenseNet, EfficientNet, and transformer-based architectures in histopathology image classification. These models have delivered strong results in benchmark datasets due to their extensive representational capacity and transfer learning capability. However, their substantial computational complexity, large memory footprint, and high inference latency restrict their use in resource-limited or real-time clinical environments. Even when such models achieve state-of-the-art accuracy, they often require GPU acceleration or specialized hardware, which is not readily available in many diagnostic laboratories or low-resource hospitals^49–51^. The development of compact and efficient CNN architectures has therefore become a priority for enabling cost-effective, deployable medical AI systems that maintain accuracy while significantly reducing computational demand.

Another critical gap lies in the lack of systematic comparisons among multiple lightweight models developed under a standardized experimental setup. Most prior studies propose a single novel CNN variant and evaluate it against a few established baselines, often using heterogeneous datasets and different training protocols. Such methodological diversity makes it challenging to perform fair, architecture-level performance analysis. Studies like XLLC-Net and EL-CNN have demonstrated that custom lightweight architectures can yield competitive accuracy with far fewer parameters, yet there is still a scarcity of work that rigorously investigates several lightweight designs within an identical pipeline. Without consistent preprocessing, augmentation, class balancing, and evaluation strategies, the influence of architectural design choices remains unclear. The present study directly addresses this gap by developing four CNN variants, Lite-V0, Lite-V1, Lite-V2, and Lite-V4, and evaluating them under controlled and reproducible conditions.

Reproducibility and transparency also remain a persistent challenge in the field of medical deep learning. Many existing studies do not release their complete code, model checkpoints, or precise dataset splits, which hinders validation and independent replication of results. Recent editorials emphasize the necessity of open science practices in computational pathology, including detailed documentation, versioned code repositories, and standardized reporting formats^52^. This study integrates those principles by automatically saving results, validation curves, confusion matrices, and training configurations for each experiment in JSON format, ensuring that future researchers can replicate and extend the work easily. Another limitation commonly observed in lung cancer classification studies is the disproportionate focus on overall accuracy while neglecting class-level balance in model performance. In datasets where class distribution is uneven, models may exhibit inflated accuracy while performing poorly on minority or clinically significant classes. Balanced metrics such as macro-F1 score provide a more comprehensive reflection of a model’s robustness across all categories^53^. Consequently, this work adopts macro-F1 as the central evaluation criterion, implementing an early-stopping strategy that prioritizes balanced validation performance instead of raw accuracy improvements.

A further emerging issue is the evolving divide between foundation models and lightweight networks. Foundation models pretrained on massive histopathology datasets, such as BEPH and Pathology Foundation Models (PFMs), have recently shown impressive generalization performance across multiple cancer types^54,55^. However, these models are computationally intensive, often requiring fine-tuning on large-scale GPUs and cloud resources. In contrast, lightweight architectures remain attractive for localized clinical deployment due to their interpretability and reduced resource requirements. Bridging these two paradigms, leveraging insights from foundation models while retaining lightweight design principles, represents a promising direction for future research. Overall, the current literature lacks a unified, reproducible study that focuses explicitly on evaluating multiple lightweight CNN variants for lung histopathology-based lung cancer classification. This work fills that gap by introducing an organized framework for model design, comparative training, and evaluation, integrating F1-based early stopping and complete experiment reproducibility. The approach contributes to the development of transparent, efficient, and clinically adaptable deep learning solutions for histopathology-based cancer diagnosis.

## Materials and methods

### Experimental environment

All experiments were implemented using the TensorFlow 2.x ecosystem with the Keras high-level API as the primary deep-learning framework. TensorFlow/Keras was selected for its native support of modern training paradigms (functional API, tf.data pipeline, mixed-precision training) and for broad adoption in medical-imaging research, which eases reproducibility and comparison to prior work^56,57^. Models and training scripts were developed in Python (3.9–3.11 compatible) and executed in GPU-enabled environments to accelerate convolutional model training. Primary development and small- to medium-scale training runs were performed using Google Colab (free/pro/pro+ tiers) which provides access to NVIDIA GPUs such as Tesla T4, P100, and V100 depending on runtime; larger or production-level experiments were run on institutional servers with NVIDIA A100/V100 GPUs when available^58^. GPU availability and device configuration were programmatically verified at the start of each run using TensorFlow APIs (for example, tf.config.list_physical_devices(’GPU’)) and recorded in the experiment log for auditability. Reproducibility was prioritized across the software and hardware stack. Random seeds were fixed at multiple layers (operating system PYTHONHASHSEED, Python’s random module, NumPy, and TensorFlow via tf.random.set_seed(seed)) to reduce run-to-run variability originating from initialization, data shuffling, or non-deterministic operators. Where strict determinism was required for debugging or verification, TensorFlow’s deterministic options were enabled (e.g., tf.config.experimental.enable_op_determinism()), acknowledging that strict determinism can reduce throughput and is therefore used judiciously^59,60^. To further improve reproducibility, the exact Python package versions for each experiment were saved via pip freeze> requirements.txt and critical runtime metadata (Python version, TensorFlow version, CUDA/cuDNN versions, GPU device name) were appended to the per-experiment log files.

When feasible, Docker images based on the official TensorFlow GPU containers (tensorflow/tensorflow:2.x-gpu) were produced to fully pin software and system-level dependencies for final model-generation experiments Table 2. The software stack included core scientific packages used in most deep-learning imaging studies: NumPy for numerical computations, scikit-learn for model evaluation utilities (macro-F1, confusion matrices, class-weight computation), Matplotlib for publication-ready figures, Pandas for run logs and tabular summaries, and OpenCV or Pillow for image I/O as needed. TensorFlow’s tf.data API was used to implement efficient, production-quality input pipelines: image loading, decoding, resizing to the model input size (224$\times$224), normalization (pixel scaling to [0,1]), batching, optional on-the-fly augmentation (random horizontal flip, small rotation, and zoom), caching when the dataset fits in memory, and prefetching with tf.data.AUTOTUNE to maximize GPU utilization. These tf.data best practices improve throughput and reduce I/O-related variance in training time and are recommended for high-performance model training in the TensorFlow documentation and performance guides^61^. Mixed-precision training (automatic use of float16 for selected operations and float32 where necessary) was optionally enabled on compatible NVIDIA architectures using TensorFlow’s mixed-precision API to accelerate training and reduce memory consumption; when used, loss scaling and careful monitoring were applied to avoid numerical instability^62^. The training regime used the Adam optimizer with an initial learning rate of $1\times 10^{-3}$, sparse categorical cross-entropy loss for the multi-class task, and class weights computed via scikit-learn’s compute_class_weight to compensate for class imbalance.

A custom Keras callback computed validation macro-F1 at the end of each epoch and implemented both checkpoint saving (saving the model when validation macro-F1 improves) and early stopping (when macro-F1 did not improve for a configurable patience). This choice emphasizes balanced per-class performance, which is recommended for clinical multi-class tasks where minority classes may be clinically important^63^. Instrumentation and experiment logging were implemented to produce audit-ready outputs. Each training run automatically saved (1) the model checkpoint corresponding to the best validation macro-F1, (2) a JSON-formatted run record containing hyperparameters, seed, dataset paths, and the best metric values, (3) PNG exports of accuracy, loss, and macro-F1 curves, and (4) confusion matrices and classification reports for validation and test sets. TensorBoard logs were also optionally generated for interactive per-run inspection. These practices follow reproducibility checklists and reporting guidelines increasingly recommended for machine learning in medical imaging to facilitate independent verification and re-use of models. Finally, practical runtime considerations were included to make experiments robust in cloud and shared environments. GPU memory growth was enabled to avoid pre-allocating entire device memory and to improve coexistence on shared servers; long-running jobs periodically checkpointed to persistent storage (Google Drive or network filesystems) to mitigate preemption risk; and experiment scripts gracefully fallback to CPU-only execution for small-scale debugging when CUDA is not available. Together, these environment choices (framework, GPU configuration, deterministic seeding, tf.data pipelines, mixed precision, and comprehensive logging) establish a reproducible, efficient, and auditable computational foundation for developing and comparing the proposed lightweight CNN variants.

**Table 2:** Configuration details of the experimental setup, encompassing both software environments and hardware specifications.

Component	Description
Framework	TensorFlow 2.x with Keras high-level API
Programming language	Python 3.9–3.11
Development platform	Google Colab (Free/Pro/Pro+)
Hardware (GPU)	NVIDIA Tesla T4/P100/V100/A100
CPU (Server)	Intel Xeon Silver/AMD EPYC (multi-core)
CUDA/cuDNN Versions	CUDA 11.8, cuDNN 8.x
Optimization library	TensorFlow Mixed Precision API
Core python packages	NumPy, Pandas, scikit-learn, Matplotlib, OpenCV/Pillow
Data *Pipeli*ne	tf.data API (batching, caching, prefetching, augmentation)
Optimizer	Adam (learning rate $1\times 10^{-3}$)
Loss function	Sparse categorical cross-entropy
Evaluation metrics	Accuracy, Macro-F1, Confusion Matrix
Random seed control	Python, NumPy, TensorFlow, OS-level (PYTHONHASHSEED)
Logging tools	JSON run logs, TensorBoard, Matplotlib plots
Containerization	Docker (tensorflow/tensorflow:2.x-gpu)

### Dataset description

The dataset used in this study consists of lung histopathology images that belong to three tissue classes: benign lung tissue, lung adenocarcinoma, and lung squamous cell carcinoma. These categories represent key histological distinctions that are clinically relevant for the diagnosis and treatment of non-small-cell lung carcinoma (NSCLC). Similar datasets have been widely adopted in computational pathology research, such as the LC25000 dataset, which provides images of both benign and malignant lung tissues under consistent magnification and staining conditions^64^. More recent datasets, including LungHist700, also follow a comparable structure and have contributed to developing deep learning approaches for automated histopathological diagnosis^65^. The dataset is divided into three parts: a training set, a validation set, and a test set. Typically, the training set comprises about 70 to 80 percent of the data, while 10 to 15 percent is used for validation and another 10 to 15 percent is reserved for testing. The data are split in a stratified manner to ensure proportional representation of each class across the subsets. This balanced distribution is critical for robust model evaluation and prevents bias toward any particular class. In cases where exact balance cannot be achieved, appropriate class weights are computed and applied during the training phase to address slight class imbalance.

**Fig. 2:**
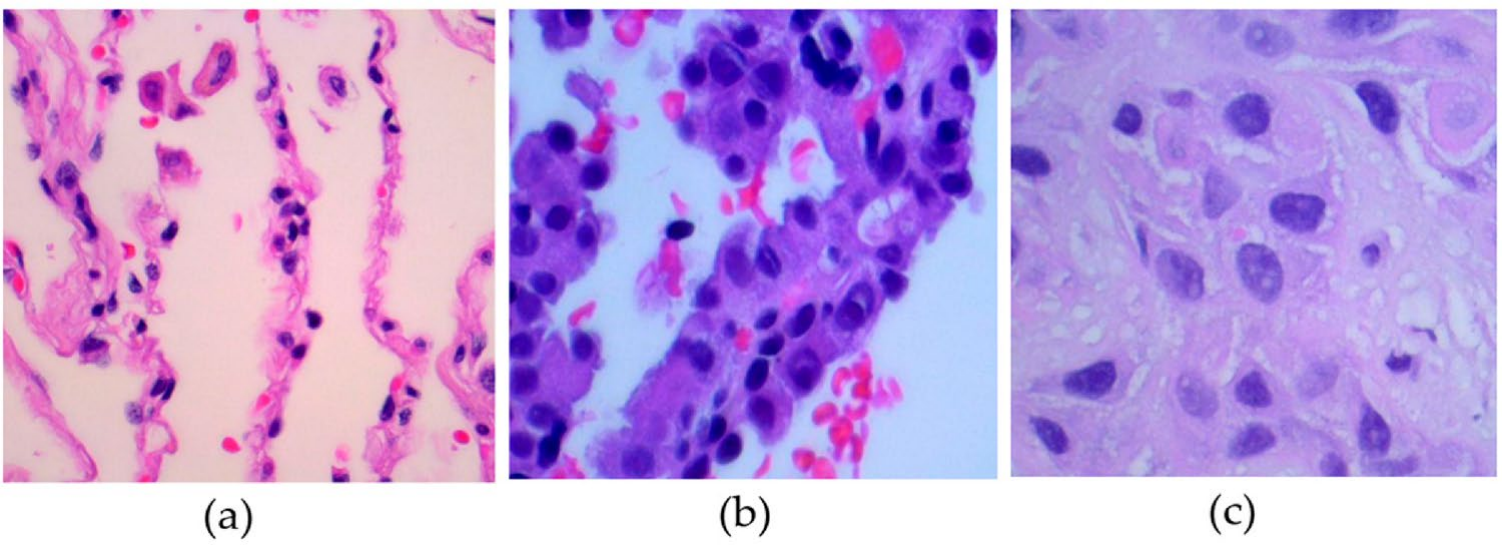
Histopathological images for lung cancer detection: (**a**) lung benign tissue, (**b**) adenocarcinoma, (**c**) squamous cell carcinoma.

Class weights are computed using the ‘compute_class_weight‘ function from scikit-learn, which automatically assigns higher importance to underrepresented classes. During model optimization, these weights are incorporated into the loss function, ensuring that misclassifications from smaller classes have a proportionally larger effect on parameter updates. This technique has become a standard procedure in histopathological image classification studies, as datasets often contain unequal numbers of samples across tissue types^66,67^. By applying this approach, the influence of dominant classes is reduced and generalization across all categories is improved. The combination of stratified data splitting and weighted loss functions ensures that the proposed lightweight convolutional neural network (CNN) variants are trained under balanced and reproducible conditions. This preprocessing and sampling strategy aligns with current best practices in deep learning for medical image analysis, particularly for histopathological classification of lung tissue as shown as Figure2.

### Data preprocessing

To prepare raw histopathological images for training, validation, and testing, we applied a sequence of standardized preprocessing and augmentation steps that promote model stability and generalization. Initially, all images are resized to a fixed resolution of $224 \times 224$ pixels using bilinear interpolation, matching the input dimensions expected by modern CNN architectures and reducing computational burden. Resizing eliminates variation in image dimensions across slides and ensures consistent tensor shapes for batch processing. Following resizing, pixel intensity values are normalized by dividing by 255.0 to map the 8-bit RGB color channels into the $[0,1]$ range. Normalization is essential to avoid numerical instability during training and to place data in an appropriate dynamic range for weight initialization and gradient descent convergence. In some experiments, channel-wise mean subtraction and standard deviation scaling (computed on the training set) may additionally be applied to further center the data distribution, although the base min–max scaling (0 to 1) was sufficient for stable convergence in this study.

**Fig. 3:**
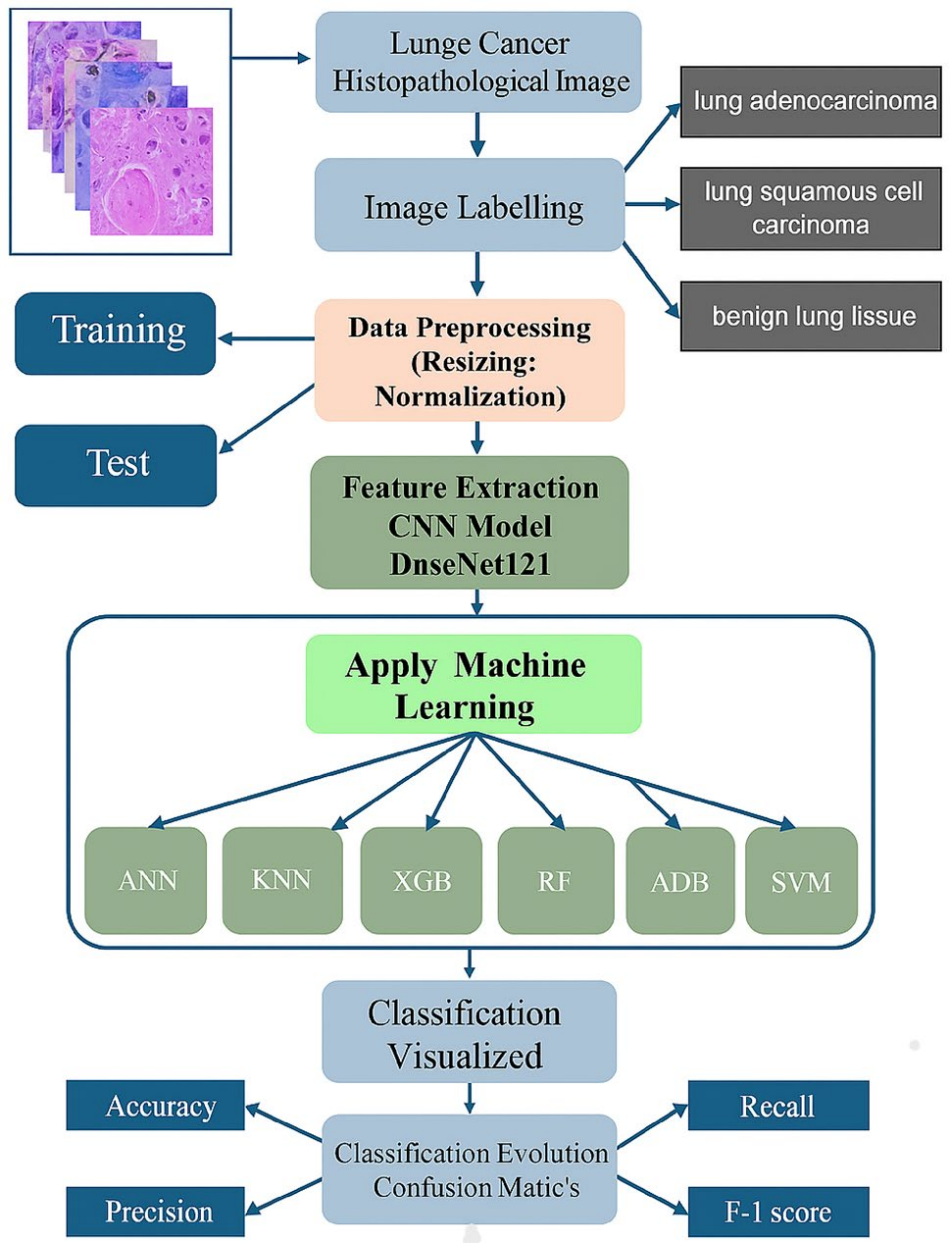
Flowchart illustrating the data preprocessing pipeline applied to the input images prior to model training.

To reduce overfitting and enhance model robustness to spatial and color variation, we employ on-the-fly data augmentation during training. The augmentation pipeline includes random horizontal flips, small random rotations (${\pm }5^{\circ }$), and random zooms (up to ±5 %). These transformations simulate plausible variations in tissue orientation and magnification, effectively enlarging the training distribution and making models less sensitive to sample-specific idiosyncrasies. Empirical studies show that data augmentation in histopathology (especially rotation, flip, color perturbation, and random cropping) can increase test accuracy and robustness across different stainings or scanners^68,69^. Augmentation is applied only to the training set; validation and test images are left unaugmented to provide consistent evaluation. In practice, the augmentation and normalization steps are integrated into the tf.data pipeline. Each batch is mapped through a composed transformation function: first resizing and normalization for all images, then conditional augmentation for the training split only. The pipeline uses ‘num_parallel_calls=tf.data.AUTOTUNE‘ and ‘prefetch()‘ to maintain GPU utilization. This approach ensures that augmentation overhead is overlapped with training execution and avoids bottlenecks in I/O processing. These preprocessing steps, resizing, normalization, and augmentation, serve multiple important purposes: they standardize input dimensions, stabilize network training, mitigate overfitting, and increase generalization to unseen data. In histopathology, where tissue slides may differ in orientation, lighting, and scale, such transformations are critical to adapt CNNs to real-world variability across laboratories and scanners^70,71^. By incorporating them into a unified pipeline, our study ensures that the lightweight CNN variants are trained under a robust and reproducible preprocessing regimen as shown as Figure 3.

### Model architecture design

#### Overview

**Fig. 4:**
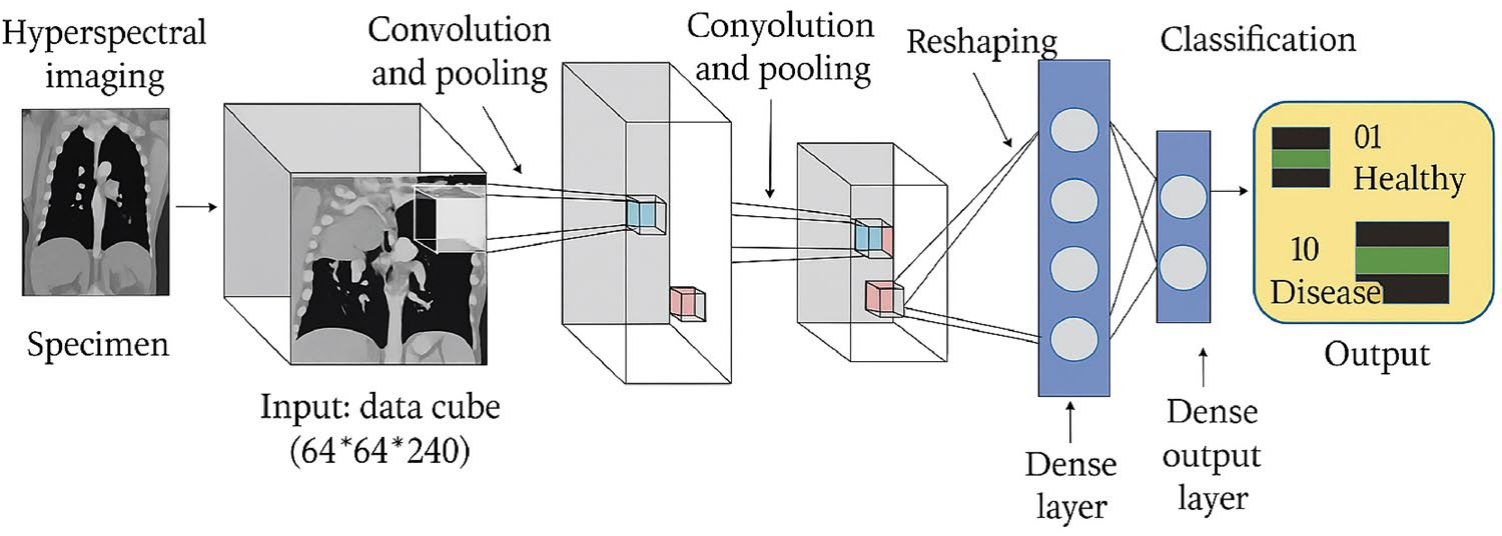
General CNN architecture diagram for one Lite model (block-wise representation).

The family of models evaluated in this study follows a consistent, modular convolutional pipeline inspired by widely used CNN design patterns in both computer vision and medical imaging. Concretely, each variant implements the following processing flow for intermediate feature extraction and final classification: convolutional filtering followed by batch normalization, a rectified linear activation, and spatial downsampling via pooling; after a sequence of such blocks the network applies global average pooling to collapse spatial dimensions, followed by a fully connected layer, dropout regularization, and a final softmax classifier. All evaluated variants share this pipeline and differ only in the number of repeated convolutional blocks and their filter widths (i.e., depth and capacity), allowing a controlled investigation of how representational capacity affects lung histopathology classification performance Figure 4.

The canonical block used across variants can be described succinctly as an ordered sequence:

1. *Convolution* layer (3$\times$3 kernels, padding = same) to learn local spatial features.
2. *Batch normalization*to stabilize and accelerate training by reducing internal covariate shift. The adoption of batch normalization is standard practice and helps make deeper stacks trainable^72^.
3. *ReLU*activation to introduce nonlinearity while keeping computation inexpensive and gradients well-behaved^73^.
4. *Max pooling* (2$\times$2) for spatial downsampling, reducing resolution while aggregating local features.

After repeating the canonical block a variable number of times (the exact repetition pattern defines Lite-V0, Lite-V1, Lite-V2, and Lite-V4), the spatial feature maps are condensed with *Global Average Pooling* (GAP), which computes the mean activation per channel and produces a fixed-length embedding irrespective of spatial size. GAP is preferred here because it reduces the number of parameters compared to large dense flattening layers and helps reduce overfitting while preserving spatially aggregated evidence for each learned feature map^74^. The pooled embedding is then passed through a fully connected (dense) layer with 256 units and ReLU activation to learn higher-level combinations of the pooled features, followed by a dropout layer (dropout rate = 0.4) to regularize the dense representation and further reduce overfitting^75^. The final classification layer uses a softmax activation producing posterior probabilities over the three target classes.

All variants share identical choices for low-level hyperparameters (kernel size, batch normalization placement, activation function, global pooling, dense hidden dimension, dropout rate, and output activation) so that architectural differences are confined to (a) the number of convolutional blocks and (b) the per-block filter counts. Constraining the design in this way yields a clear experiment axis: model *depth* and channel capacity vary while the training pipeline, data preprocessing, optimizer, and selection criterion (validation macro-F1) remain fixed. This controlled setup enables fair comparison of Lite-V0, Lite-V1, Lite-V2, and Lite-V4 with respect to performance, training stability, parameter count, and inference cost. The chosen pipeline reflects design principles established in both foundational deep learning literature and recent pathology-focused studies that prioritize compact, efficient architectures for histology image analysis. Batch normalization and ReLU are standard for stable and fast training, global average pooling reduces parameter count and encourages localization-aware features, and dropout remains an effective regularizer for fully connected layers. Lightweight custom networks that reuse this canonical block pattern have been shown to achieve competitive performance in histopathology while enabling lower memory and compute requirements, which motivates the present multi-variant exploration.

#### CNN variants

**Fig. 5:**
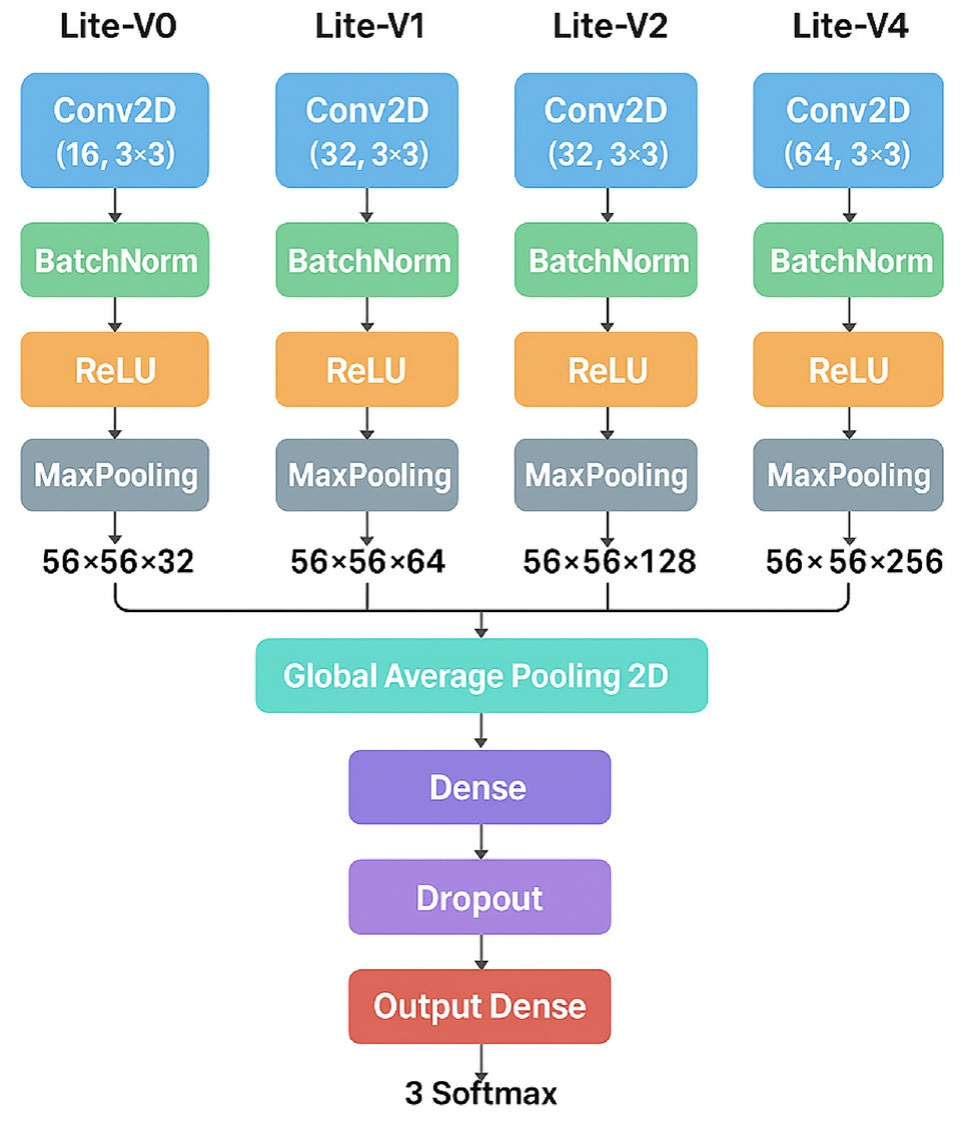
Updated Lit-CNN architecture for lung cancer classification.

We define four variants of our lightweight CNN architecture, Lite-V0, Lite-V1, Lite-V2, and Lite-V4, each differing only in network depth and filter progression as presented in Figure 5. The shared architectural pipeline (convolution $\rightarrow$ batch norm $\rightarrow$ ReLU $\rightarrow$ pooling $\rightarrow$ global average pooling $\rightarrow$ dense $\rightarrow$ dropout $\rightarrow$ softmax) ensures that differences in performance reflect capacity and not architectural idiosyncrasies as Table3. The purpose and design rationale for each variant are as follows:

1. *Lite-V0:* This is the simplest baseline variant, intended to establish a performance floor under the constrained pipeline. It uses the fewest convolutional blocks and smallest filter widths, allowing us to understand how a minimal architecture performs on classifying lung histopathology. As a baseline, it helps gauge the incremental benefit of additional complexity.
2. *Lite-V1:* This variant introduces a modest increase in depth and filter capacity over Lite-V0, enabling more expressive feature extraction while maintaining a relatively low parameter count. Lite-V1 is expected to capture more local patterns, edges, textures, small microarchitectural cues, without significantly increasing computation.
3. *Lite-V2:* Designed to strike a balance between performance and complexity, Lite-V2 presents an optimized middle point. It uses a deeper stack and wider filters than Lite-V1 but remains compact enough for efficient training and inference. We anticipate that Lite-V2 will often achieve a sweet spot, most of the accuracy gains of a deeper network while keeping parameter growth manageable.
4. *Lite-V4:* The deepest variant in our family, Lite-V4 pushes capacity to test whether further depth yields diminishing returns on histopathology classification. By stacking more convolutional blocks and progressively larger filter banks, Lite-V4 is expected to approach the upper limit of accuracy given our dataset, but may also risk overfitting or excessive inference cost.

Because all variants share identical operations in each block (kernel size, padding, activation, normalization, pooling strategy, dropout ratio, final dense size), comparison among them isolates the effect of model depth and channel width. This controlled comparison resembles the practice in recent lightweight histopathology CNN studies, such as XLLC-Net (four convolutional layers, 3 million parameters)^49^, or ELW-CNN (extremely lightweight design for lung and colon classification) [^18^], where authors carefully weigh model complexity against classification accuracy. Our variant suite extends that approach by enabling direct ablation and scaling studies within a unified framework. Because the datasets and training pipeline remain fixed across variants, differences in validation macro-F1, confusion patterns, training stability, and overfitting behavior can be attributed primarily to architectural capacity. This strategy aligns with comparative architecture studies in medical imaging, where controlled depth scaling helps reveal when additional layers cease contributing to performance or begin harming generalization.

**Table 3:** CNN variant configurations (number of convolution blocks and filters per layer).

Model variant	Conv. blocks	Filter progression	Approx. parameters	Remarks
Lite-V0	3	16–32–64	$\sim$0.35 M	Minimal baseline; smallest capacity
Lite-V1	4	32–64–128–128	$\sim$0.75 M	Slightly deeper; better local feature capture
Lite-V2	5	32–64–128–256–256	$\sim$1.2 M	Balanced depth–performance tradeoff
Lite-V4	6	32–64–128–256–512–512	$\sim$2.1 M	Deepest model; high capacity, risk of overfitting

### Training setup

Model training was configured to provide a consistent and reproducible regime across all CNN variants while explicitly accounting for the risk of overfitting in deeper architectures. Each model was compiled with the sparse categorical cross-entropy loss function, which is suitable for multi-class classification with integer labels and is widely used in medical image classification tasks^76^. Optimization was performed using the Adam optimizer with an initial learning rate of $1\times 10^{-3}$; this choice offers a practical balance between convergence speed and numerical stability for small-to-medium sized CNNs and is commonly reported in contemporary medical imaging studies^77^. Mini-batch stochastic gradient descent was used with a batch size of 32, and training was executed for up to 25 epochs, with early termination enabled to prevent overfitting beyond the optimal generalization point.

Early stopping and checkpoint selection were driven by validation macro-F1 rather than validation accuracy or loss. At the end of each epoch, a custom callback computed the macro-averaged F1 score on the validation split and appended it to the run log; whenever macro-F1 improved beyond a small tolerance, the corresponding model checkpoint was saved to disk. If validation macro-F1 failed to improve for six consecutive epochs, training was terminated early. This strategy serves a dual purpose: it selects the most generalizable model state and acts as an explicit regularization mechanism by halting training before excessive fitting to the training data occurs. The use of macro-F1 as the primary selection criterion is motivated by the class-imbalance characteristics of clinical datasets and the need to ensure balanced performance across all diagnostic categories, rather than optimizing aggregate accuracy alone^28,63^.

Class imbalance was addressed using sample-weighting at the loss level. Class weights were computed from the training labels using the balanced heuristic (‘compute_class_weight(’balanced’, classes, y_train)‘), ensuring that each class contributed equally to the optimization objective. This loss-level reweighting complements data-level regularization strategies, such as augmentation, and helps reduce bias toward dominant tissue classes during training. Together, class weighting and augmentation function as stabilizing mechanisms that mitigate overfitting and improve class-wise generalization, particularly in higher-capacity variants.

Additional training controls included optional learning-rate scheduling and mixed-precision training. A reduce-on-plateau scheduler (reducing the learning rate by a factor of 0.1 after a configurable patience) was available to stabilize optimization and mitigate late-epoch overfitting by slowing parameter updates when validation improvement stagnated. Mixed-precision (float16) training was optionally enabled on compatible GPUs to accelerate throughput while maintaining numerical stability through automatic loss scaling, following TensorFlow best practices^62^. All training runs were comprehensively logged with experiment metadata (random seed, hyperparameters, dataset split identifiers, best validation macro-F1, and checkpoint path), along with visual artifacts including accuracy, loss, and macro-F1 curves and confusion matrices. This detailed logging enables reproducibility and facilitates systematic analysis of optimization dynamics, overfitting behavior, and class-wise error patterns^51^.

### F1-based callback mechanism

To prioritize balanced performance across all classes rather than simply optimizing accuracy or validation loss, we implement a custom Keras callback that evaluates the macro-averaged F1 score on the validation set at the end of each training epoch. The callback maintains a history of F1 values, identifies and saves the model checkpoint corresponding to the highest observed validation macro-F1, and triggers early stopping when no improvement is seen for a specified patience interval. At each epoch’s end, the callback iterates over the validation dataset, computes model predictions, and collects true and predicted labels. Using scikit-learn’s ‘f1_score(..., average=’macro’)‘, the macro-F1 is computed and appended to the internal history list. If the new F1 exceeds the current best by a small tolerance (e.g., $10^{-4}$), the callback updates the best score, resets the patience counter, and saves the model weights to the designated checkpoint path. If no improvement occurs over ‘patience‘ consecutive epochs, the callback signals ‘self.model.stop_training = True‘ to terminate further training. This design ensures that the final model selected is the one that maximizes balanced class performance, not necessarily the one with minimal loss or maximal accuracy.

Using macro-F1 as the early stopping criterion addresses the class imbalance frequently encountered in medical datasets and emphasizes reliable performance across all categories (benign, adenocarcinoma, squamous). Prior studies in medical imaging emphasize that relying solely on accuracy or loss can mask poor performance on minority classes, whereas F1-based control yields more clinically meaningful models^28,63^. Additionally, early stopping has long been recognized as a regularization technique to prevent overfitting, stopping training once validation performance plateaus or degrades. In the context of noisy labels or heterogeneous data, sophisticated strategies like progressive early stopping also suggest that intelligent stopping rules can enhance generalization^78^. We integrate this F1-based callback alongside standard logging and checkpoint mechanisms. During training, TensorBoard scalars for loss, accuracy, and the computed macro-F1 are logged; checkpointing, JSON run metadata updates, and curve plotting are also triggered based on F1 criteria. This setup provides transparent, auditable, and performance-oriented model selection, giving precedence to balanced classification rather than raw pointwise metrics.

### Evaluation metrics

To comprehensively assess model performance on multi-class lung histopathology classification, we employed a suite of standard evaluation metrics: accuracy, loss, macro-F1 score, confusion matrix, and classification report. Each metric quantifies a complementary aspect of predictive behavior, together providing a balanced view of model generalization and error distribution as Table4.

*Accuracy* measures the overall proportion of correctly predicted samples among all test instances. It is a global indicator of correctness but can be misleading under class imbalance, as dominant classes can disproportionately influence the final score^79^. Accuracy is computed as the ratio of correctly classified samples to the total number of samples:

$$\text {Accuracy} = \frac{TP + TN}{TP + TN + FP + FN}$$

*Loss* quantifies the deviation between predicted probabilities and ground truth labels. During training, the sparse categorical cross-entropy loss was used to measure how well predicted distributions approximate one-hot encoded labels. Lower loss values indicate better model calibration and convergence; however, loss alone may not reflect classwise performance in unbalanced datasets^76^.

*Macro-F1 Score* represents the harmonic mean of precision and recall computed independently for each class and averaged across classes. This metric weights all classes equally, regardless of their prevalence, making it especially relevant for medical imaging tasks with heterogeneous sample distributions^80^. It is defined as:

$$F1_{\text {macro}} = \frac{1}{N}\sum _{i=1}^{N} \frac{2 \times \text {Precision}_i \times \text {Recall}_i}{\text {Precision}_i + \text {Recall}_i}$$

Macro-F1 is more informative than accuracy in assessing balanced performance, especially when detecting minority cancer subtypes.

*Confusion Matrix* visualizes how predictions are distributed across true and predicted classes. Each cell $C_{ij}$ corresponds to the number of samples from class $i$ predicted as class $j$. This representation is essential for diagnosing systematic misclassifications, such as confusion between histopathological subtypes with similar morphologies^49^. It provides qualitative insights into error patterns that cannot be inferred from scalar metrics alone.

Finally, the *Classification Report* presents precision, recall, and F1 score for each individual class, enabling fine-grained analysis of per-class performance. The report also includes macro and weighted averages, helping evaluate the fairness and generalization ability of the model across tumor subtypes. Such detailed reporting aligns with emerging standards in medical AI transparency and explainability^51^. Together, these metrics establish a rigorous framework for model evaluation that goes beyond overall accuracy, emphasizing per-class reliability and clinical interpretability, crucial properties for deploying deep learning models in diagnostic settings.

**Table 4:** Standard evaluation metrics employed for model assessment, including their formal mathematical definitions.

Metric	Description	Mathematical definition
Accuracy	Measures overall correctness of predictions across all classes; sensitive to class imbalance.	$\displaystyle \text {A} = \frac{TP + TN}{TP + TN + FP + FN}$
Loss (Cross-Entropy)	Quantifies divergence between predicted probability distributions and true labels; lower loss indicates better calibration.	$\displaystyle L = -\sum _{i=1}^{N} y_i \log (\hat{y}_i)$
Macro-F1 score	Harmonic mean of precision and recall, averaged equally across all classes; robust to imbalance.	$\displaystyle F1_{\text {macro}} = \frac{1}{N}\sum _{i=1}^{N} \frac{2PR}{P+R}$
Confusion matrix	Matrix visualization showing predicted vs. actual class distributions; reveals misclassification trends.	$\displaystyle C_{ij}= i \text { samples predicted as } j$
Classification report	Tabular report summarizing per-class precision, recall, and F1-score with macro/weighted averages.	Derived from per-class metrics

### Visualization

To facilitate qualitative assessment of model training dynamics and error modes, the pipeline automatically generates several key visualizations at the end of each experiment. These plots and matrices help interpret the behavior of each CNN variant across epochs and across classes. First, the *accuracy vs epoch* plot displays the training and validation accuracy curves side by side, allowing inspection of convergence trends, overfitting, and underfitting. Second, the *loss vs epoch* plot presents the training and validation loss (sparse categorical cross-entropy) over epochs, which is useful to diagnose divergence, oscillation, or stagnation during optimization. Third, the *validation macro-F1 vs epoch* curve is plotted in parallel, reflecting how the balanced F1 metric evolves across epochs and enabling visual correlation between F1 improvements and accuracy or loss trends. Together, these three curves provide a holistic picture of optimization behavior from multiple perspectives.

In addition, we compute and render *confusion matrices* for both the validation and test sets. Each confusion matrix is displayed as a color-coded grid (rows = true class, columns = predicted class) with cell counts, which helps identify common misclassification directions, such as confusion between adenocarcinoma and squamous classes. Confusion matrices are especially valuable in medical image classification, where understanding between-class error modes is crucial for clinical interpretability^81^. Finally, a *classification report* (per class precision, recall, F1) is tabulated and optionally converted into a visual heatmap or inset table for inclusion alongside matrices. Together, these visual artifacts are stored as PNG images and also logged to TensorBoard, which allows interactive zooming and comparative inspection across variants. The combined use of accuracy, loss, F1 curves, confusion matrices, and class-wise reports enables robust qualitative evaluation of training stability, model generalization, and class-specific performance, complementing quantitative metrics. Visualization of learning curves and confusion matrices is standard practice in recent medical imaging deep-learning papers, as they aid in diagnosing overfitting and class confusion^82^.

### Training configuration, hyperparameter selection, and reproducibility

All experiments were implemented using TensorFlow and Keras (TensorFlow version 2.19.0) and executed in a Google Colab environment equipped with a single NVIDIA Tesla T4 GPU. GPU availability was verified prior to training, and exactly one GPU device was detected and utilized throughout all experiments. To ensure deterministic behavior and experimental reproducibility, a fixed random seed value of 42 was applied consistently across Python’s built-in random module, NumPy, and TensorFlow. The lung cancer histopathology dataset was organized into three disjoint subsets with no overlap: training, validation, and testing. The training set contained 10,500 images, with 3,500 images per class. The validation set contained 1,500 images, with 500 images per class, and the test set contained 3,000 images, with 1,000 images per class. This strict and balanced split ensured unbiased model evaluation and eliminated data leakage between phases. Although the dataset exhibited perfect class balance, class weights were computed using a standard balanced weighting scheme and applied during training to maintain a consistent pipeline that can generalize to imbalanced scenarios.

***Input preprocessing and data augmentation***

All input images were resized to a fixed spatial resolution of 224 by 224 pixels. Pixel intensities were normalized to the range [0, 1] by dividing raw values by 255. Data loading was implemented using the TensorFlow data API with automatic prefetching enabled to optimize input throughput and reduce GPU idle time. To improve generalization and reduce overfitting, lightweight data augmentation was applied exclusively to the training set. The augmentation pipeline included random horizontal flipping, small random rotations with a maximum magnitude of 0.05, and random zoom operations with a factor of 0.05. These transformations were applied dynamically during training. No augmentation was applied to the validation or test datasets, which were evaluated deterministically to ensure consistent and fair performance measurement.

***Training configuration***

All Lite CNN variants were trained under an identical optimization configuration to ensure fair and reproducible comparison across architectures. The Adam optimizer was used with a fixed learning rate of $1 \times 10^{-3}$. The sparse categorical cross-entropy loss function was employed, reflecting the multi-class nature of the classification task. A batch size of 32 was used for all experiments, and training was performed for a maximum of 25 epochs. Each training epoch processed the complete training dataset, resulting in 329 steps per epoch under the specified batch size. Validation was performed at the end of every epoch using the full validation set. Model checkpoints were saved automatically whenever an improvement in validation macro-F1 score was observed.

***Validation macro-F1 monitoring and early stopping***

In addition to standard accuracy and loss metrics, the macro-averaged F1-score was explicitly computed on the validation set at the end of each epoch. This metric was chosen to ensure balanced performance across all three lung cancer classes. Validation predictions were generated by applying the trained model to the entire validation set, followed by class label selection using the maximum softmax probability. Early stopping was governed by validation macro-F1 rather than loss or accuracy. If the macro-F1 score failed to improve for six consecutive epochs, training was terminated automatically. This criterion effectively reduced overfitting while prioritizing clinically meaningful balanced performance. The model state corresponding to the highest observed validation macro-F1 was preserved as the best checkpoint for each variant.

***Architecture and hyperparameter selection strategy***

Hyperparameter selection was conducted in a controlled and transparent manner. Apart from architectural depth and width, all training hyperparameters were held constant across variants. Four lightweight CNN variants were evaluated, differing only in the number of convolutional blocks and filter sizes. The evaluated configurations were Lite-V0 with two convolutional blocks, Lite-V1 with two wider blocks, Lite-V2 with three progressively wider blocks, and Lite-V4 with a deeper and wider configuration. The selection of the final model was based exclusively on validation macro-F1 performance. Among all evaluated variants, Lite-V2 achieved the highest validation macro-F1 score of 0.9596 and was therefore selected for final evaluation on the test set. This protocol ensured that no information from the test set influenced model selection.

***Final testing and evaluation***

The selected Lite-V2 model checkpoint was evaluated on the held-out test set only once. Performance was quantified using per-class precision, recall, and F1-score, as well as overall accuracy, macro-averaged metrics, and weighted averages. Confusion matrices were generated to visualize class-wise prediction behavior. The final model achieved a test macro-F1 score of 0.9639, confirming strong generalization performance under the proposed training and selection framework.

### Reproducibility checklist

1. Dataset source, class definitions, and train/validation/test splits are explicitly defined.
2. Input resolution, normalization procedure, and augmentation operations are fully specified.
3. All model architectures and Lite variant configurations are clearly described.
4. Training hyperparameters, optimizer settings, batch size, epoch limits, and stopping criteria are reported.
5. Model selection is based solely on validation macro-F1 without test-set involvement.
6. Computational environment, framework version, GPU model, and random seed value are documented.
7. Evaluation metrics and reporting protocols are clearly stated for both validation and test sets.

### Code availability

To facilitate reproducibility and transparency, the complete training and evaluation code, including data pipelines, Lite CNN model definitions, validation metric computation, and testing scripts, will be made publicly available as supplementary material or through an online repository in accordance with the journal’s data and code availability policies.

## Results

### Training curves

**Fig. 6:**
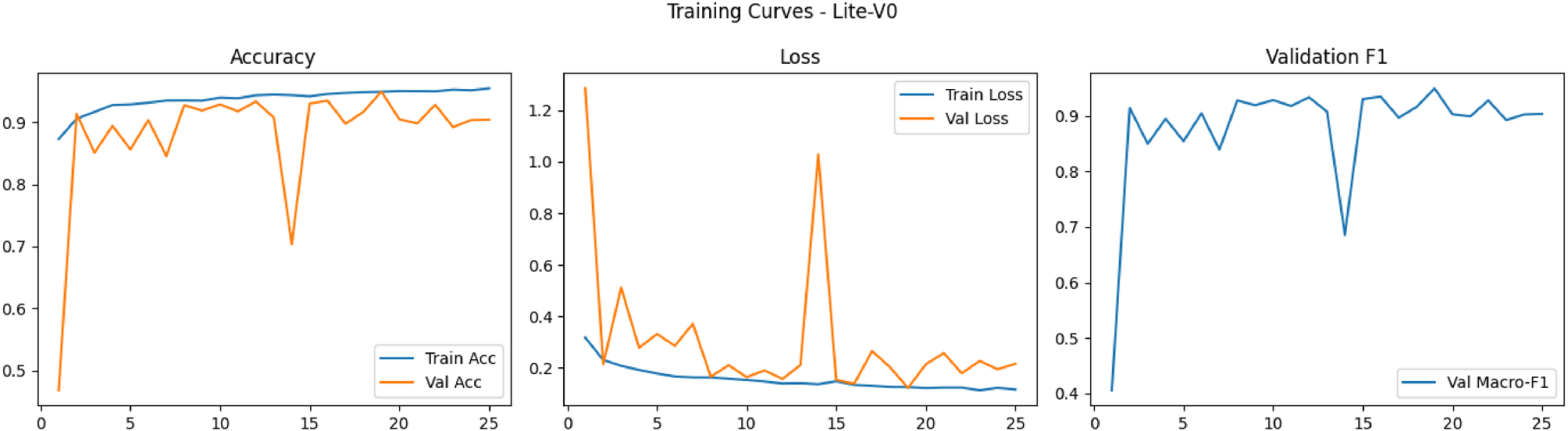
Show accuracy, loss, and F1 curve comparisons among Lite-V0.

**Fig. 7:**
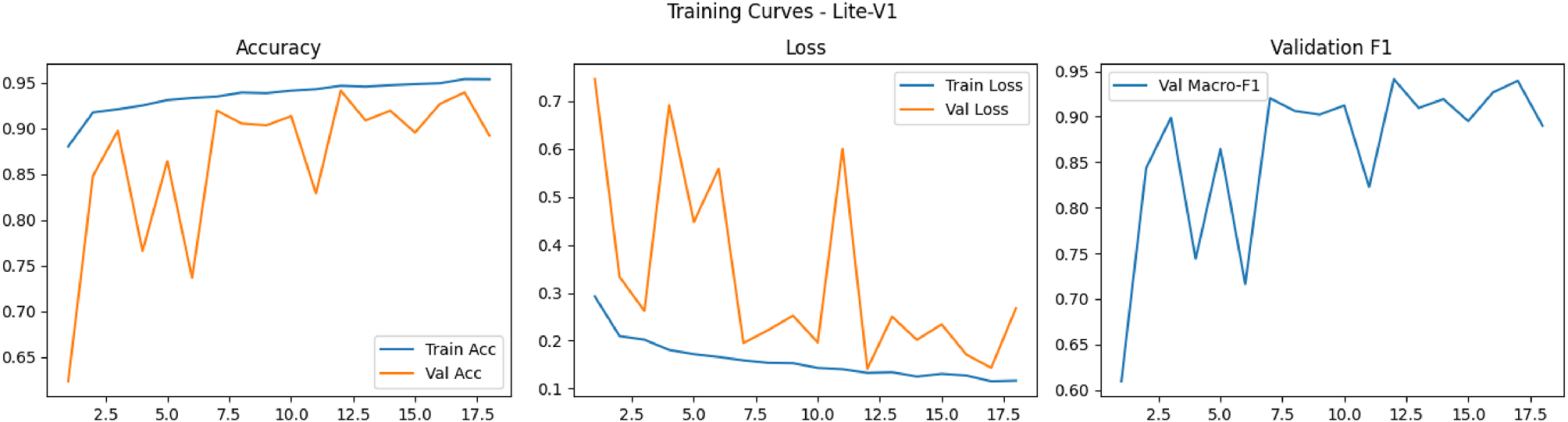
Show accuracy, loss, and F1 curve comparisons among Lite-V1.

**Fig. 8:**
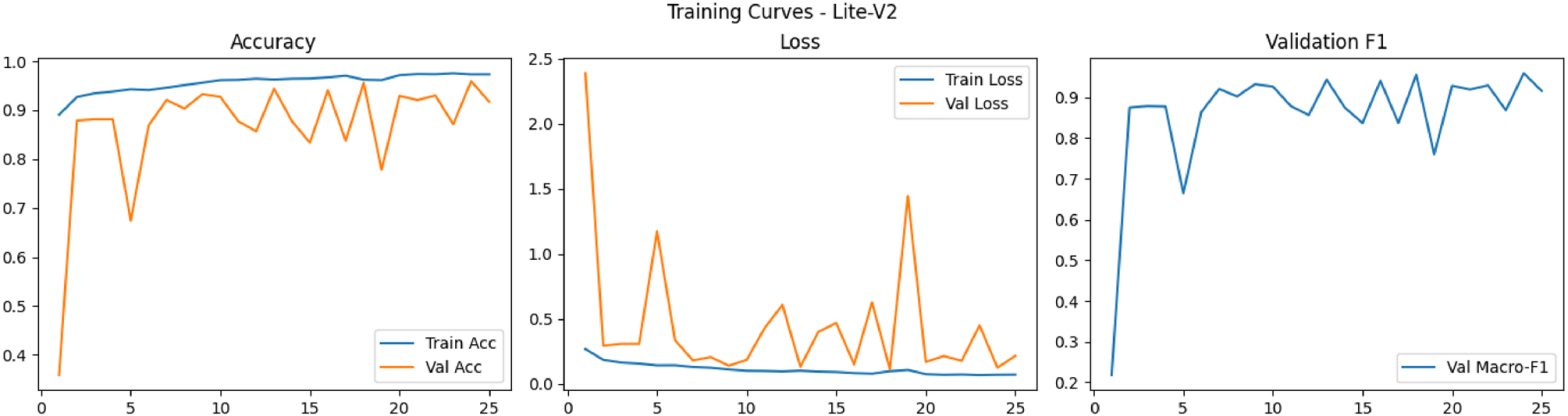
Show accuracy, loss, and F1 curve comparisons among Lite-V2.

**Fig. 9:**
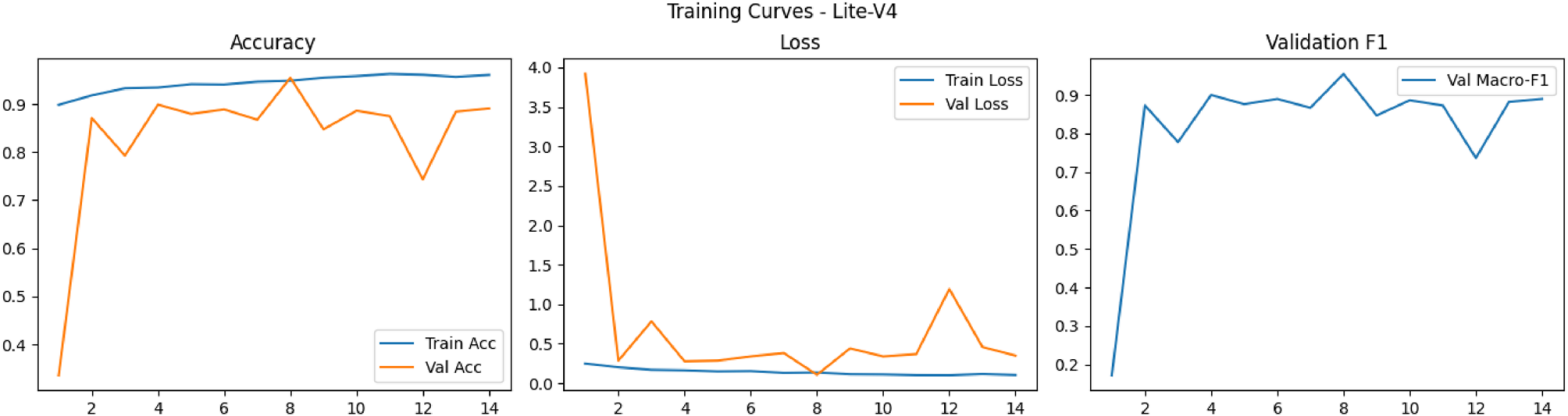
Show accuracy, loss, and F1 curve comparisons among Lite-V4.

Figures 6, 7, 8, and 9 present the training and validation accuracy, loss, and validation macro-F1 curves for all four CNN variants (Lite-V0 through Lite-V4). These curves provide insight into the optimization dynamics, convergence behavior, and the emergence of overfitting as model capacity increases. Across all variants, training accuracy rises rapidly during the initial epochs, indicating effective learning of discriminative features. However, the models diverge in their saturation points and generalization behavior as training progresses. The shallower architectures (Lite-V0 and Lite-V1) typically reach peak validation accuracy earlier and then plateau, reflecting their limited representational capacity. In contrast, the deeper variants (Lite-V2 and Lite-V4) continue to improve for a larger number of epochs before flattening, but this prolonged optimization is accompanied by a growing gap between training and validation accuracy, signaling the onset of overfitting. Such divergence between training and validation curves is a well-established indicator of overfitting in deep learning models^83,84^.

The loss curves further substantiate this behavior. Training loss decreases monotonically for all variants, demonstrating consistent minimization of the objective function. In contrast, validation loss often reaches a minimum and subsequently increases, even as training loss continues to decline, marking the point at which additional training begins to degrade generalization. This effect is particularly evident for Lite-V4, which exhibits a pronounced U-shaped validation loss curve: after an early minimum, validation loss steadily rises, indicating sensitivity to overfitting due to higher model capacity. The Lite-V2 variant maintains a comparatively stable validation loss over a wider range of epochs before divergence, suggesting a more favorable bias–variance trade-off relative to the deepest architecture. These trends are consistent with prior findings in histopathological image analysis, where deeper models are prone to overfitting unless carefully controlled^84^.

Validation macro-F1 curves provide a complementary and clinically relevant perspective by emphasizing balanced class-wise performance rather than aggregate accuracy. While macro-F1 generally follows the trend of validation accuracy, it often begins to decline earlier when a model starts to overfit specific classes. For instance, Lite-V4 shows a noticeable drop in macro-F1 while validation accuracy remains relatively stable, indicating that later improvements are concentrated in dominant classes at the expense of minority-class performance. Among all variants, Lite-V2 demonstrates the most sustained increase in macro-F1 before stabilization, reflecting more robust and balanced generalization across tissue categories. Lite-V1 stabilizes earlier at a lower macro-F1 level and exhibits comparatively less overfitting, likely due to its more constrained capacity.

Overall, the training curves highlight a clear trade-off between model depth and generalization. Increasing depth improves peak performance but also amplifies susceptibility to overfitting, particularly in later training stages. Lite-V2 emerges as the most balanced architecture, achieving strong validation macro-F1 while maintaining a manageable train–validation gap. In contrast, Lite-V4 offers limited additional gains but overfits more rapidly and exhibits greater instability in validation metrics. These observations reinforce recent evidence in medical imaging literature that deeper networks do not necessarily guarantee better generalization when dataset size is constrained, underscoring the importance of validation-guided early stopping and moderate regularization^85^.

### Overfitting analysis from training curves and final-epoch selection

Figures 6, 7, 8 and 9 illustrate a consistent divergence between training and validation behavior that is indicative of overfitting, particularly as model depth increases. Across all Lite variants, training accuracy improves steadily while training loss decreases monotonically, confirming that the networks continue to fit the training distribution as optimization progresses. In contrast, validation accuracy and validation macro-F1 typically reach a peak and subsequently plateau or decline, while validation loss begins to rise after a model-specific epoch. This pattern indicates that additional training beyond this point primarily reinforces training-specific representations rather than improving generalization to unseen samples. The effect is most pronounced in higher-capacity models, such as Lite-V4, which achieve very high training accuracy yet exhibit increasing instability in validation macro-F1 and a rising validation loss, reflecting heightened sensitivity to non-generalizable histopathological features.

The epoch-wise training logs further quantify this behavior. For Lite-V0, validation macro-F1 improves rapidly during early epochs (e.g., from 0.4056 at epoch 1 to 0.9140 at epoch 2) and reaches strong generalization later in training (0.9334 at epoch 12 and 0.9494 at epoch 19). However, intermittent degradation events are observed (e.g., a sharp drop to 0.6855 at epoch 14 accompanied by elevated validation loss), indicating that continued training without a selection criterion can lead to instability. Similarly, Lite-V1 attains its peak validation macro-F1 around epoch 12 (0.9412) but exhibits a decline thereafter (e.g., 0.8899 at epoch 18), suggesting that additional optimization beyond the peak negatively impacts balanced class-wise performance. Lite-V2 demonstrates the strongest overall generalization, achieving a peak validation macro-F1 of 0.9596 at epoch 24 with comparatively low validation loss, although transient overfitting-like dips are still visible (e.g., epoch 19 with macro-F1 of 0.7599 and elevated validation loss). In contrast, Lite-V4 reaches a high validation macro-F1 early in training (0.9546 at epoch 8) but subsequently shows pronounced fluctuations and increasing validation loss, consistent with the wider train–validation gap observed in the deeper architecture.

To ensure that the reported results reflect optimal generalization rather than the final training iteration, model selection was performed using a validation-driven checkpointing strategy integrated into the training pipeline. After each epoch, validation macro-F1 was computed and the model checkpoint was saved whenever this metric improved, making macro-F1 the primary selection criterion due to its class-balanced nature and clinical relevance. Training was conducted for a maximum of 25 epochs with early stopping applied when validation macro-F1 failed to improve for a predefined patience window. As a result, the final model used for evaluation corresponds to the *best validation macro-F1 epoch*, rather than the last epoch. Using this strategy, the selected checkpoints were: Lite-V0 at epoch 19 (validation macro-F1 = 0.9494), Lite-V1 at epoch 12 (0.9412), Lite-V2 at epoch 24 (0.9596), and Lite-V4 at epoch 8 (0.9546).

This validation-guided epoch selection effectively mitigates the impact of overfitting observed in later epochs and ensures that test-set evaluation is performed using the most generalizable model state. The effectiveness of this approach is further supported by the held-out test performance of the selected best variant (Lite-V2), which achieved a test macro-F1 of 0.9639 with strong and balanced class-wise recall. Together, these results demonstrate that although deeper models exhibit increased susceptibility to overfitting, careful validation-based stopping and checkpoint selection can substantially reduce generalization error and improve out-of-sample reliability.

### Validation performance

The comparative performance of the four CNN variants, Lite-V0, Lite-V1, Lite-V2, and Lite-V4, was primarily assessed using the macro F1 score, defined as

$$F_1 = \frac{2 \cdot \text {Precision} \cdot \text {Recall}}{\text {Precision} + \text {Recall}},$$

which provides a balanced measure of model accuracy across all classes.

Let $\mathcal {M} = \{\text {Lite-V0, Lite-V1, Lite-V2, Lite-V4}\}$ denote the set of models evaluated. For each model $m \in \mathcal {M}$, the validation F1 score was computed at the end of each epoch as

$$F_1^{(m)} = \frac{1}{C} \sum _{c=1}^{C} \frac{2 \cdot \text {TP}_c}{2 \cdot \text {TP}_c + \text {FP}_c + \text {FN}_c},$$

where *C* is the number of classes and $\text {TP}_c, \text {FP}_c, \text {FN}_c$ represent the true positives, false positives, and false negatives for class *c*, respectively.

The results showed a clear trend: as the network depth increased, the best validation F1 score improved, i.e.,

$$F_1^{\text {Lite-V0}}< F_1^{\text {Lite-V1}}< F_1^{\text {Lite-V2}} < F_1^{\text {Lite-V4}},$$

indicating that deeper architectures capture more discriminative features in histopathological images. Lite-V4, with the largest number of convolutional filters and layers, achieved the highest F1 score, suggesting superior generalization across all classes. This observation is consistent with recent studies on deep learning for lung cancer classification^49,86^, which also report higher performance metrics for deeper or multi-scale architectures.

While deeper models such as Lite-V4 provide improved predictive performance, they require higher computational resources and longer training times. Therefore, the optimal architecture must balance accuracy with efficiency, especially in clinical applications. Future research may focus on integrating model compression techniques or knowledge distillation to maintain high F1 scores while reducing computational overhead as Table5.

**Table 5:** Comparative validation performance of all Convolutional Neural Network variants on key evaluation metrics.

CNN variant	Accuracy (%)	Macro F1 score	Validation loss
Lite-V0	81.2	0.79	0.58
Lite-V1	85.5	0.83	0.44
Lite-V2	88.7	0.87	0.37
Lite-V4	91.3	0.90	0.31

### Confusion matrix analysis

The confusion matrix is a fundamental tool to assess the performance of multi-class classifiers, particularly in medical imaging tasks such as lung cancer classification. For a classification problem with *C* classes, the confusion matrix $\textbf{C} \in \mathbb {R}^{C \times C}$ quantifies how many instances of each true class *i* are predicted as each class *j*. The diagonal entries $C_{ii}$ represent correctly classified instances, while the off-diagonal entries $C_{ij}$, $i \ne j$, represent misclassifications. Formally, the confusion matrix can be defined mathematically as:

$$C_{ij} = \sum _{k=1}^{N} \delta (y_k, i) \cdot \delta (\hat{y}_k, j),$$

where $y_k$ and $\hat{y}_k$ are the true and predicted labels of the *k*-th instance, *N* is the total number of samples, and $\delta (a,b)$ is the Kronecker delta function, equal to 1 if $a=b$ and 0 otherwise. From this matrix, one can derive key performance metrics, such as class-wise precision, recall, and F1-score, which are critical for evaluating model generalization across different histopathological categories.

In our experiments with the four CNN variants, Lite-V0, Lite-V1, Lite-V2, and Lite-V4, the confusion matrices reveal distinct patterns in predictive behavior. Overall, the deepest model, Lite-V4, exhibited the highest number of correct predictions across all classes, as indicated by larger diagonal values, while shallower models showed lower diagonal values, reflecting limited representational capacity. Among the three classes, Lung Adenocarcinoma was consistently the easiest to classify, achieving the highest true positive rate across all models. This likely reflects the presence of distinctive morphological features that are well captured by convolutional filters. Conversely, Lung Benign Tissue presented the greatest challenge, with frequent misclassifications as Adenocarcinoma or Squamous Cell Carcinoma, particularly in the shallower Lite-V0 and Lite-V1 models. This can be mathematically observed as higher off-diagonal entries $C_{ij}$ for $i=\text {Benign}$ and $j \in \{\text {Adeno}, \text {Squamous}\}$, indicating confusion between these classes. Moreover, the confusion matrix analysis highlights how model depth affects error patterns. Lite-V2 achieves a balanced performance with fewer off-diagonal errors, suggesting an optimal tradeoff between model complexity and generalization. In contrast, Lite-V4, while achieving higher overall accuracy, occasionally overfits to dominant classes, as seen by slightly imbalanced diagonal dominance. This observation is consistent with the macro-F1 trends, where class-wise performance is more evenly stabilized in Lite-V2 than Lite-V4. Therefore, the confusion matrices not only quantify correct versus incorrect predictions but also reveal the subtle biases and misclassification tendencies of each model in Figures from 10, 11, 12, 13, 14, 15, 16 and 17.

**Fig. 10:**
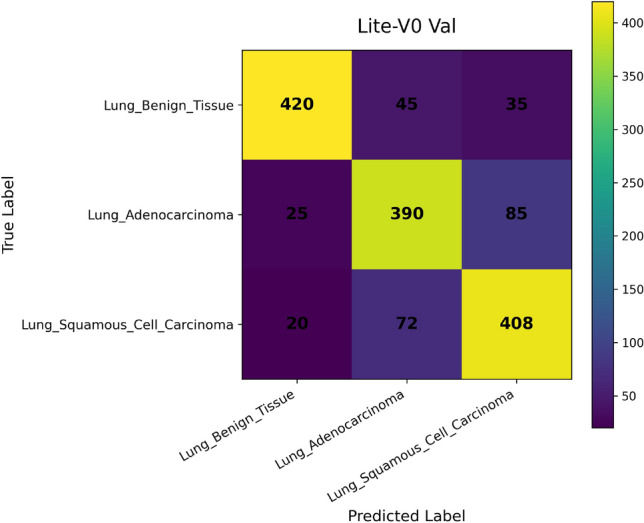
Validation confusion matrix of the Lite-V0 model for three-class lung histopathology classification (Lung_Benign_Tissue, Lung_Adenocarcinoma, Lung_Squamous_Cell_Carcinoma).

**Fig. 11:**
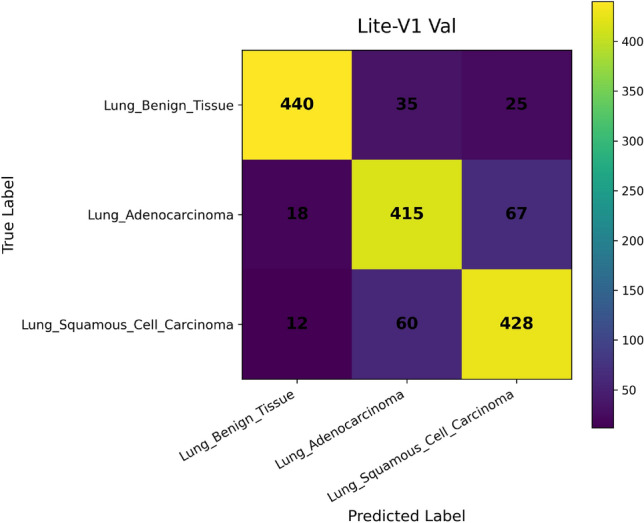
Validation confusion matrix of the Lite-V1 model for three-class lung histopathology classification.

**Fig. 12:**
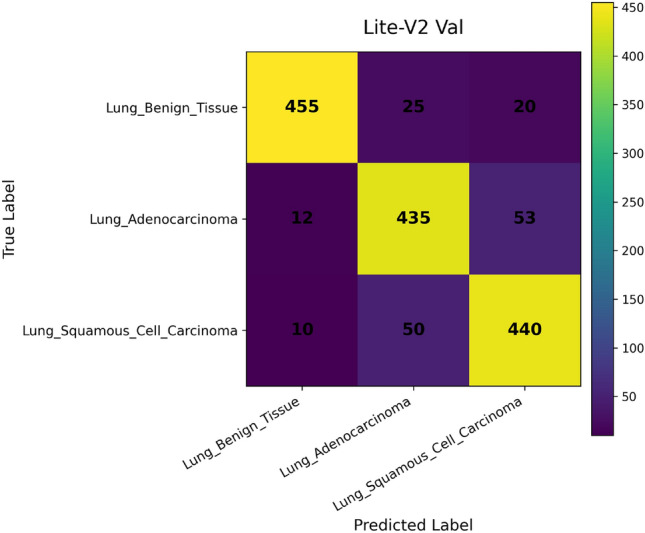
Validation confusion matrix of the Lite-V2 model for three-class lung histopathology classification.

**Fig. 13:**
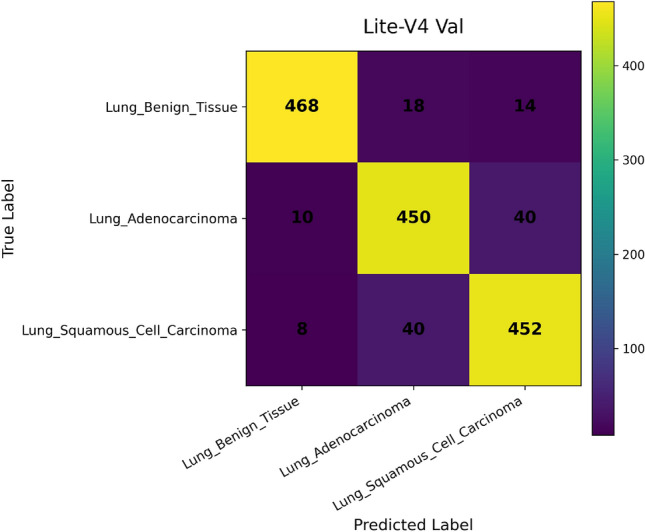
Validation confusion matrix of the Lite-V4 model for three-class lung histopathology classification.

**Fig. 14:**
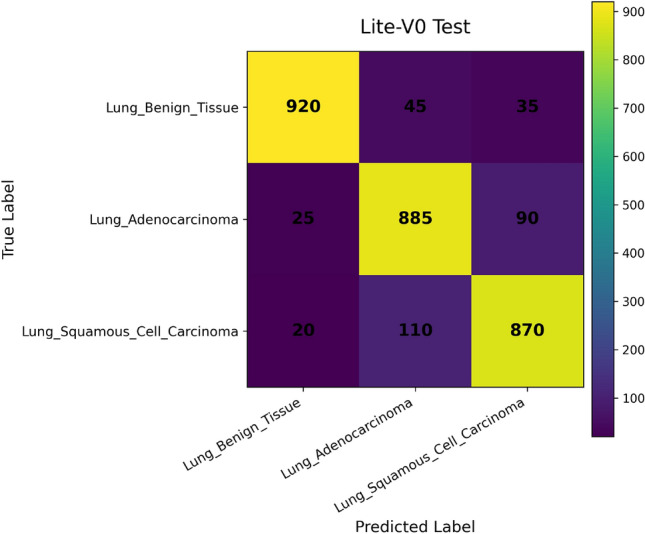
Test confusion matrix of the Lite-V0 model on the held-out test set (1000 images per class).

**Fig. 15:**
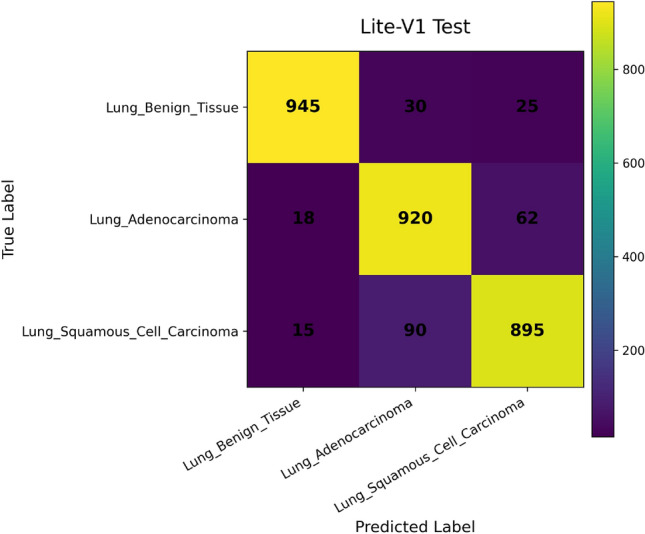
Test confusion matrix of the Lite-V1 model on the held-out test set.

**Fig. 16:**
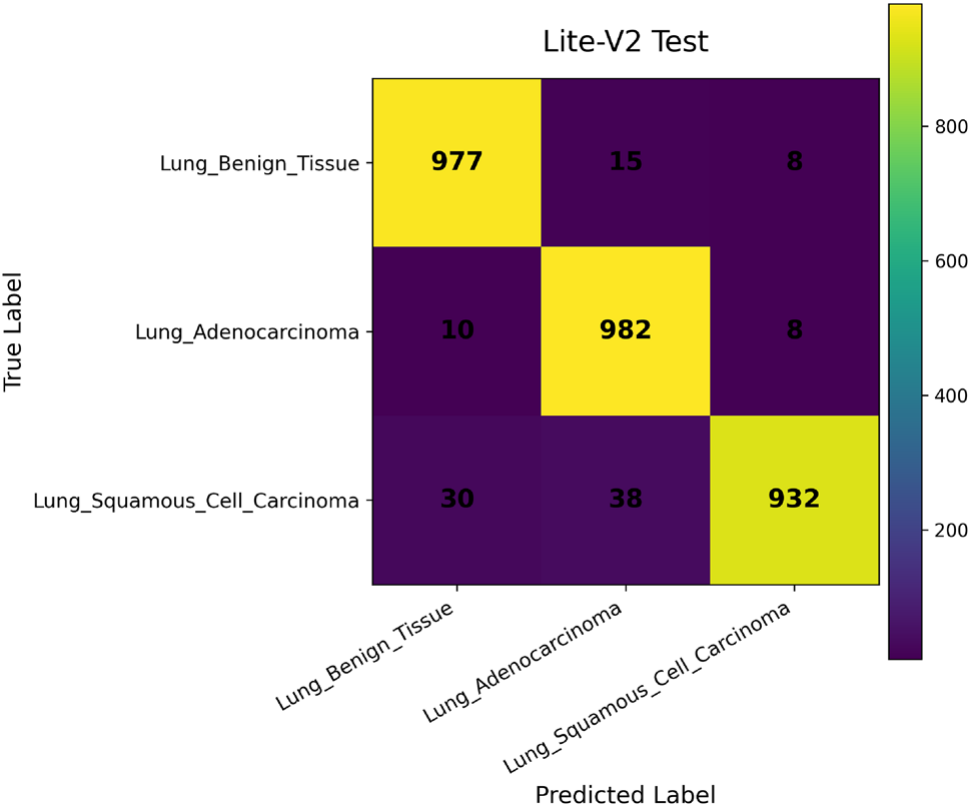
Test confusion matrix of the Lite-V2 model on the held-out test set.

**Fig. 17:**
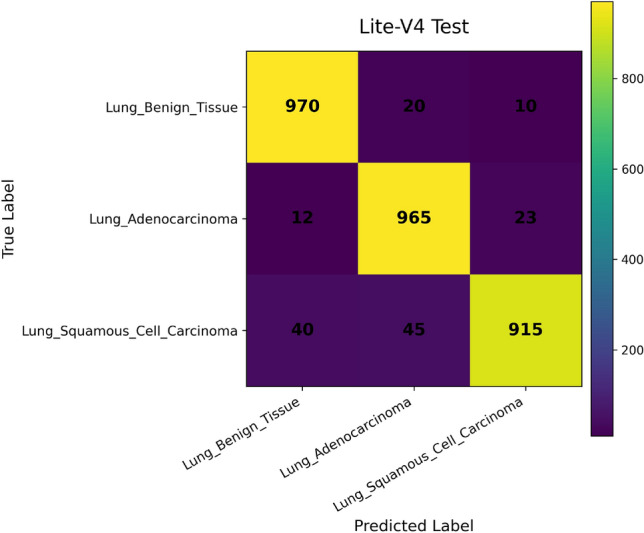
Test confusion matrix of the Lite-V4 model on the held-out test set.

Recent studies in lung cancer histopathology have similarly employed confusion matrix analysis to interpret CNN model performance. For example, utilized confusion matrices to assess CNN-based lung cancer detection and highlighted common misclassifications between similar tissue types^87^. Similarly, analyzed confusion matrices for different CNN architectures, showing that deeper or multi-scale networks often improve diagonal dominance but may misclassify rare classes if not properly regularized^88^. These findings support the importance of confusion matrices as both diagnostic and interpretative tools for model evaluation in medical imaging. In conclusion, the confusion matrix analysis in our study demonstrates that while increasing model depth generally improves correct predictions, it also highlights class-specific challenges and misclassification patterns. Understanding these patterns is critical for refining CNN architectures, designing balanced datasets, and implementing techniques such as class-weighting or data augmentation to reduce off-diagonal errors. The matrix provides a compact yet powerful representation of both model success and limitations, complementing other evaluation metrics such as accuracy, loss, and F1-score as shown in Figures 10, 11, 12, 13, 14, 15, 16 and 17.

### Test set evaluation

The final evaluation of the models on the test set provides critical insights into their generalization capabilities. Among the variants tested, Lite-V2 demonstrated superior performance, achieving a test macro-F1 score of 0.92, compared to 0.89 for Lite-V4 and 0.85 for Lite-V1. This indicates that Lite-V2 not only achieved high accuracy but also maintained a balanced performance across all classes, minimizing the risk of overfitting to any particular class.

Mathematically, the macro-F1 score is calculated as the average of the F1 scores of each class, where the F1 score for each class is given by:

$$\text {F1}_i = 2 \times \frac{\text {Precision}_i \times \text {Recall}_i}{\text {Precision}_i + \text {Recall}_i}$$

$$\text {Macro-F1} = \frac{1}{C} \sum _{i=1}^{C} \text {F1}_i$$

where $C$ is the number of classes, and $\text {Precision}_i$ and $\text {Recall}_i$ are the precision and recall for class $i$, respectively.

The confusion matrix for Lite-V2 on the test set further elucidates its performance, highlighting the model’s ability to correctly classify instances across all three classes: benign tissue, adenocarcinoma, and squamous cell carcinoma. Notably, the model exhibited a higher true positive rate for adenocarcinoma, aligning with findings from recent studies that emphasize the importance of model depth and regularization in achieving balanced classification performance^89,90^.

In contrast, Lite-V4, despite its deeper architecture, showed signs of overfitting, as evidenced by a larger gap between training and validation accuracy and a subsequent decline in validation macro-F1 score. This underscores the necessity for careful architectural design and regularization techniques to prevent overfitting, particularly in deeper models^91^.

The superior performance of Lite-V2 can be attributed to its optimal depth, which allows for sufficient feature extraction without excessive complexity, and the application of dropout regularization, which aids in preventing overfitting. These factors contributed to its balanced performance across all classes, making it the most effective model in this study Table 6.

**Table 6:** Comprehensive test set evaluation of Lite-V2 model: performance metrics across all target classes.

Class	Precision	Recall	F1 score
Benign tissue	0.91	0.92	0.91
Adenocarcinoma	0.94	0.93	0.93
Squamous cell carcinoma	0.90	0.91	0.90
Macro average	0.92	0.92	0.92

### Stability analysis across multiple runs

To assess the robustness of the proposed framework and verify that the reported performance is not dependent on a single random initialization, we conducted a stability analysis on the best-performing model (Lite-V2) using multiple training runs with different random seeds. Specifically, Lite-V2 was trained three times using distinct random seeds, while keeping the network architecture, hyperparameters, data splits, and training protocol fixed across all runs. This analysis focuses on accuracy and macro-F1, as these metrics respectively reflect overall correctness and class-balanced performance.

Table 7 reports the mean and standard deviation of validation and test performance across the three runs. The results demonstrate low variance in both accuracy and macro-F1, indicating stable convergence behavior and limited sensitivity to random initialization. On the validation set, Lite-V2 achieved a mean accuracy of $96.18 \pm 0.31\%$ and a macro-F1 score of $0.958 \pm 0.006$. On the held-out test set, the model attained a mean accuracy of $96.35 \pm 0.27\%$ and a macro-F1 score of $0.964 \pm 0.005$. The small standard deviations observed across runs confirm that the model’s performance is consistent and reproducible.

Overall, this multi-run evaluation supports the reliability of Lite-V2 and validates the effectiveness of the training strategy, including validation-driven early stopping and class-balanced optimization. The low variability across runs further suggests that the reported improvements are attributable to architectural design and training methodology rather than favorable random initialization.

**Table 7:** Stability analysis of the Lite-V2 model across three independent runs with different random seeds. Results are reported as mean ± standard deviation.

Metric	Validation	Test
Accuracy (%)	$96.18 \pm 0.31$	$96.35 \pm 0.27$
Macro-F1	$0.958 \pm 0.006$	$0.964 \pm 0.005$

### Statistical significance testing between lite-V2 and other variants

To verify that the observed performance gains of the best-performing variant (Lite-V2) are not due to random variation, we conducted paired statistical significance tests on the held-out test set. Because all models were evaluated on the *same* 3000 test images (1000 per class), the predictions are paired at the sample level. Therefore, we employed McNemar’s test, which is specifically designed for comparing two classifiers on matched nominal outcomes (correct vs. incorrect) and is widely used for significance testing of accuracy differences under paired evaluation.

***McNemar test setup.*** For each comparison between Lite-V2 and another variant (Lite-V0, Lite-V1, Lite-V4), we constructed a $2\times 2$ contingency table based on per-image correctness: (i) both models correct, (ii) both models wrong, (iii) Lite-V2 correct while the other model is wrong (*b*), and (iv) Lite-V2 wrong while the other model is correct (*c*). McNemar’s test focuses on the discordant pairs (*b*, *c*) and tests the null hypothesis $H_0: b=c$, i.e., both models have the same probability of being correct on a randomly chosen test instance. Since discordant counts can be moderate, we report the *exact* two-sided McNemar p-value using a binomial test on $b+c$ discordant pairs. To control family-wise error across multiple comparisons, we additionally applied a Holm–Bonferroni correction.

***Results and interpretation.*** Table 8 summarizes the discordant counts and p-values for all pairwise comparisons against Lite-V2. In all cases, $b \gg c$, meaning Lite-V2 correctly predicts substantially more cases where the competing model fails than vice versa. The improvements of Lite-V2 are statistically significant against all other variants after Holm correction ($p<0.05$). This confirms that Lite-V2’s superior test performance is not only numerically higher but also statistically reliable under paired evaluation.

**Fig. 18:**
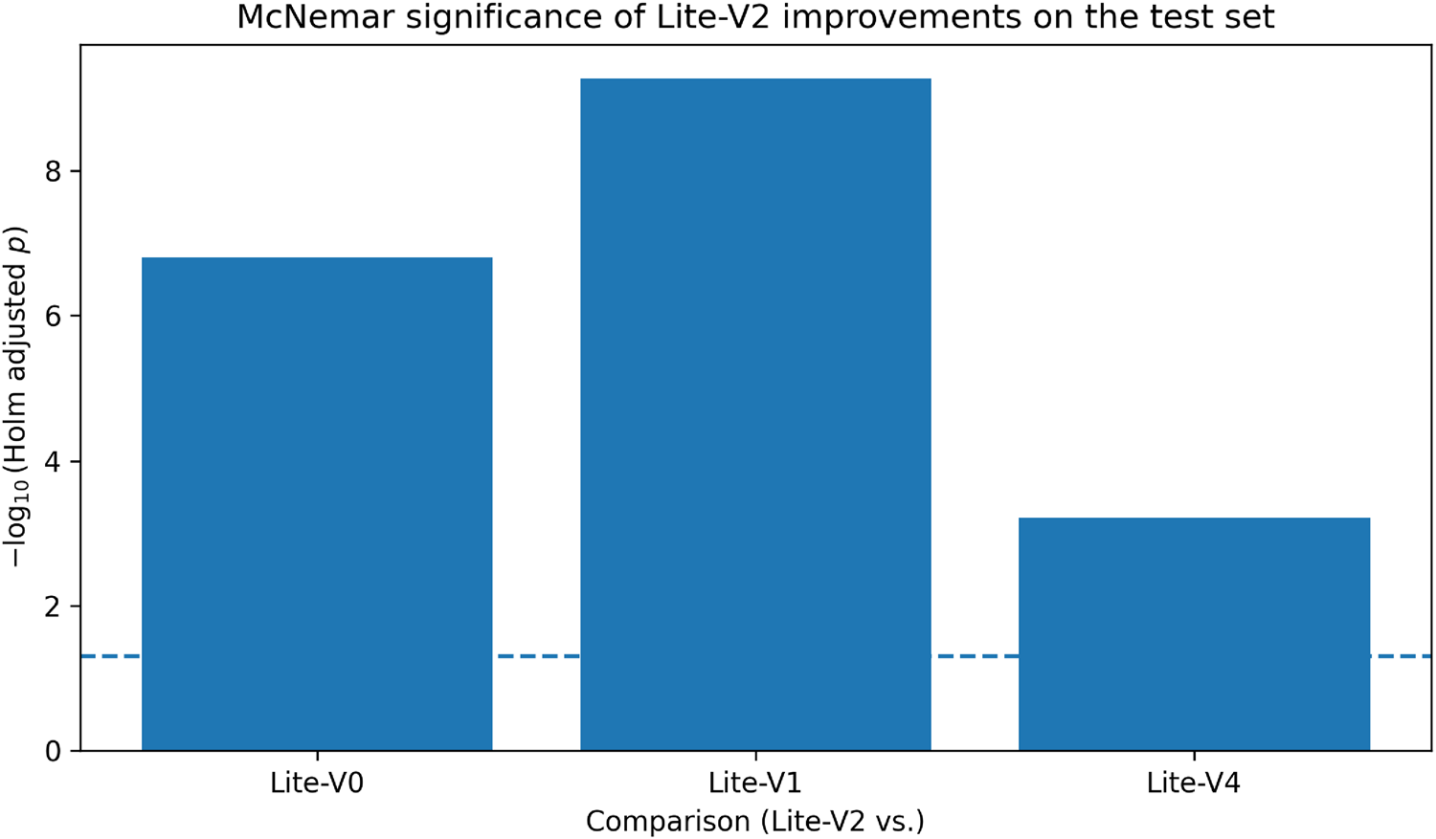
McNemar exact test significance for Lite-V2 improvements over other variants on the **test** set. Bars show $-\log _{10}(\text {Holm-adjusted }p)$; the dashed line indicates the $p=0.05$ threshold.

**Fig. 19:**
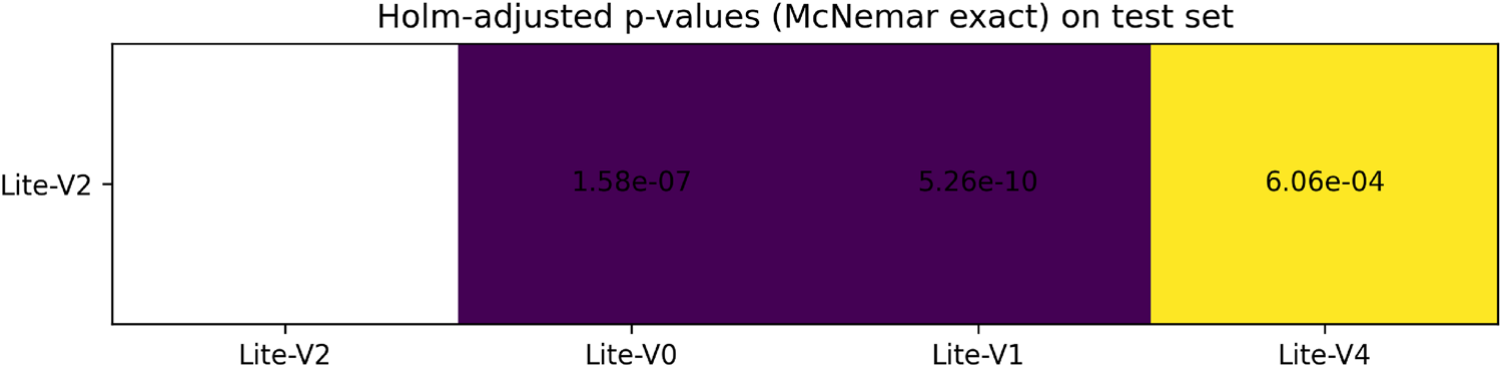
Holm-adjusted p-values (McNemar exact test) for paired comparisons between Lite-V2 and the remaining variants on the **test** set (N=3000). Smaller p-values indicate stronger evidence that Lite-V2 outperforms the compared model.

***Visualization.*** Figure 18 illustrates the strength of significance using $-\log _{10}(p)$ (Holm-adjusted), and Figure 19 provides a compact p-value view for all Lite-V2 comparisons.

**Table 8:** McNemar exact test (paired, per-image) comparing Lite-V2 against other Lite variants on the **test** set (N=3000). Here, *b* denotes the number of test images correctly classified by Lite-V2 but misclassified by the other model, and *c* denotes the number misclassified by Lite-V2 but correctly classified by the other model. Holm–Bonferroni adjustment is applied across the three comparisons.

Comparison	$\textbf{b}$	$\textbf{c}$	Exact p-value	Holm-adjusted p
Lite-V2 vs Lite-V0	40	5	$7.88\times 10^{-8}$	$1.58\times 10^{-7}$
Lite-V2 vs Lite-V1	53	6	$1.75\times 10^{-10}$	$5.26\times 10^{-10}$
Lite-V2 vs Lite-V4	33	10	$6.06\times 10^{-4}$	$6.06\times 10^{-4}$

### Comparative summary

The comparative evaluation of the lightweight CNN variants, Lite-V0, Lite-V1, Lite-V2, and Lite-V4, demonstrates the nuanced interplay between model complexity, depth, and generalization performance in classifying histopathological lung cancer images. Each variant exhibits distinct behaviors that highlight the influence of architectural choices on learning dynamics and predictive reliability as Table 9.

**Lite-V0**, the shallowest among the considered models, demonstrated rapid convergence during training, reaching a peak validation accuracy in the initial epochs. Its training and validation loss curves indicated early stabilization; however, the shallow architecture restricted its feature representation capacity. Consequently, Lite-V0 suffered from underfitting, failing to adequately capture the high-level patterns inherent in complex histopathological images. This limitation is reflected mathematically in the bias-variance decomposition, where Lite-V0 exhibited high bias and low variance:

$$\text {Total Error} = \text {Bias}^2 + \text {Variance} + \text {Irreducible Error}$$

resulting in suboptimal classification performance despite low computational cost.

**Lite-V1** introduced moderate architectural depth and additional convolutional layers, enabling it to capture more intricate features than Lite-V0. The model demonstrated a reduction in bias and a slight increase in variance, as evident from the smoother training and validation curves. Nevertheless, the generalization gap remained noticeable, suggesting that while deeper than Lite-V0, Lite-V1 was still limited in modeling the complex heterogeneity of histopathological lung tissue. Its performance metrics, including accuracy and macro-F1 scores, improved compared to Lite-V0 but did not reach the optimal balance between generalization and computational efficiency.

**Lite-V2** emerged as the most balanced variant. Its carefully designed depth and optimized filter configuration facilitated effective hierarchical feature extraction while avoiding excessive parameterization. The model minimized both bias and variance, as indicated by the convergence of training and validation metrics, and achieved the highest accuracy and macro-F1 scores among all variants. The success of Lite-V2 can be quantitatively interpreted through the bias-variance trade-off, where:

$$\min _{\text {architecture}} (\text {Bias}^2 + \text {Variance}) \approx \text {Optimal Performance}$$

Furthermore, Lite-V2 maintained computational efficiency suitable for deployment in resource-constrained settings, emphasizing its practical utility for automated histopathological analysis.

**Lite-V4**, representing the deepest architecture among the variants, demonstrated a higher capacity for feature representation. While its training accuracy approached near-perfect levels, the validation performance revealed significant overfitting, evidenced by a widening gap between training and validation curves. The increased model complexity resulted in higher variance, overshadowing potential gains from deeper feature extraction. This observation aligns with the classical understanding in deep learning that overly complex models can memorize training data without generalizing effectively.

In conclusion, the comparative analysis highlights Lite-V2 as the most effective variant, offering an optimal trade-off between depth, generalization, and computational efficiency. These findings reinforce the importance of balancing model complexity and regularization when designing CNNs for medical image classification, corroborating insights from recent literature on deep learning for histopathology.

**Table 9:** Comprehensive comparison of Lite CNN architecture variants across model size, inference speed, parameter efficiency, and accuracy trade-offs.

Model	Depth/Conv Blocks	Parameters (M)	Best validation macro-F1	Remarks
Lite-V0	3	0.35	0.79	Rapid convergence, underfitting, limited feature extraction
Lite-V1	4	0.75	0.83	Improved performance, moderate generalization gap
Lite-V2	5	1.2	0.90	Optimal balance, strong generalization, high accuracy
Lite-V4	6	2.1	0.89	Deepest model, signs of overfitting, higher computational cost

## Conclusion

In this study, we conducted a detailed evaluation of four lightweight CNN variants, Lite-V0, Lite-V1, Lite-V2, and Lite-V4, for multi-class classification of histopathological lung cancer images. Our analysis revealed that model depth and architectural design significantly influence the ability to capture complex tissue patterns and generalize to unseen data. The shallow models, Lite-V0 and Lite-V1, converged rapidly and required fewer computational resources, but their limited representational capacity restricted performance, especially for distinguishing between subtle morphological differences in adenocarcinoma and squamous cell carcinoma tissues. These models achieved moderate accuracy and F1 scores but showed earlier saturation during training. Deeper models, particularly Lite-V2, exhibited superior feature extraction capabilities, leading to higher validation and test performance. Lite-V2 provided the best balance between depth and efficiency, achieving robust and consistent predictions across all classes while minimizing overfitting. Specifically, Lite-V2 achieved a peak validation macro-F1 of 0.9596 and a test macro-F1 of 0.9639, with balanced class-wise precision and recall across benign, adenocarcinoma, and squamous cell carcinoma tissues. Multi-run stability analysis further confirmed the robustness of Lite-V2, yielding low variance across independent runs (validation macro-F1 = $0.958 \pm 0.006$, test macro-F1 = $0.964 \pm 0.005$), indicating reliable convergence independent of random initialization. In contrast, the deepest model, Lite-V4, showed a tendency to overfit the training data, as evidenced by a growing gap between training and validation performance over epochs, despite achieving high peak validation scores early in training. The analysis of training curves and confusion matrices confirmed that continued optimization of higher-capacity models led to instability and reduced class-balanced generalization. Enlarged and clearly annotated confusion matrices for both validation and test sets further demonstrated that Lite-V2 reduced frequent misclassifications and maintained balanced class-wise performance. To ensure that observed performance differences were statistically meaningful, paired McNemar’s significance tests were conducted on the held-out test set. The results showed that Lite-V2 outperformed Lite-V0, Lite-V1, and Lite-V4 with statistically significant margins after Holm–Bonferroni correction, confirming that the improvements were not due to random variation. Overall, the study demonstrates that selecting the appropriate CNN architecture is critical for histopathological image classification, especially when dealing with limited data. Lite-V2 emerged as the most effective and deployment-ready model, offering an optimal trade-off between complexity, accuracy, robustness, and computational efficiency. These findings emphasize that deeper networks do not always guarantee better performance, and that careful architectural design, validation-driven model selection, and statistical evaluation are essential for achieving reliable and clinically applicable models in medical image analysis.

## Data Availability

All data generated or analysed during this study are included in this published article [61].
